# A cooperative PNPase-Hfq-RNA carrier complex facilitates bacterial riboregulation

**DOI:** 10.1016/j.molcel.2021.05.032

**Published:** 2021-07-15

**Authors:** Tom Dendooven, Dhriti Sinha, Alzbeta Roeselová, Todd A. Cameron, Nicholas R. De Lay, Ben F. Luisi, Katarzyna J. Bandyra

**Affiliations:** 1 Department of Biochemistry, University of Cambridge, Tennis Court Road, Cambridge CB2 1GA, UK; 2 Department of Microbiology & Molecular Genetics, University of Texas Health Science Center, 6431 Fannin Street, MSE R266, Houston, TX 77030, USA

**Keywords:** small regulatory RNA, riboregulation, RNA chaperone, ribonuclease, polynucleotide phosphorylase, Hfq, gene silencing, ribonucleoprotein complex, cryoEM

## Abstract

Polynucleotide phosphorylase (PNPase) is an ancient exoribonuclease conserved in the course of evolution and is found in species as diverse as bacteria and humans. Paradoxically, *Escherichia coli* PNPase can act not only as an RNA degrading enzyme but also by an unknown mechanism as a chaperone for small regulatory RNAs (sRNAs), with pleiotropic consequences for gene regulation. We present structures of the ternary assembly formed by PNPase, the RNA chaperone Hfq, and sRNA and show that this complex boosts sRNA stability *in vitro.* Comparison of structures for PNPase in RNA carrier and degradation modes reveals how the RNA is rerouted away from the active site through interactions with Hfq and the KH and S1 domains. Together, these data explain how PNPase is repurposed to protect sRNAs from cellular ribonucleases such as RNase E and could aid RNA presentation to facilitate regulatory actions on target genes.

## Highlights

• Cryo-EM structures of PNPase in complex with the RNA chaperone Hfq and regulatory RNA
• Structural insights into regulatory RNA recognition by Hfq and PNPase
• Model for stabilization of regulatory RNAs and facilitation of their functions

## Graphical abstract

**Figure:**
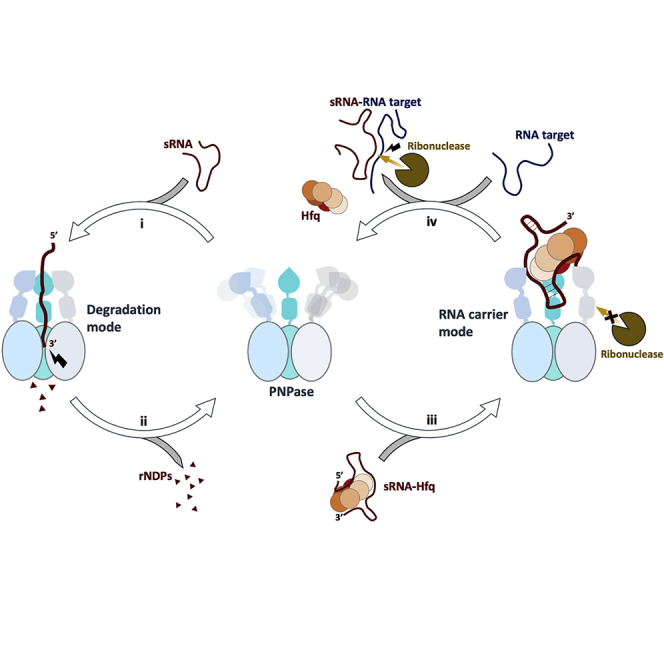

## Teaser

The conserved exoribonuclease PNPase contributes to RNA turnover in many organisms, but in bacteria the enzyme can be re-programmed by the RNA chaperone Hfq and regulatory RNA to switch from degradative to chaperoning roles in RNA-mediated gene regulation. Dendooven et al. provide structural insight into the basis for this functional switch and details of the recognition of complex regulatory RNAs by Hfq and PNPase.

## Introduction

In all domains of life, ribonucleases are key players in post-transcriptional regulation of gene expression. These enzymes catalyze mRNA degradation and the maturation of rRNA and tRNA precursors, and they often have an impact on the stability of regulatory RNAs (Deutscher, 2015). One of the key ribonucleases in diverse organisms is polynucleotide phosphorylase (PNPase), an exoribonuclease of ancient evolutionary origin that contributes to RNA degradation and RNA quality control (Danchin, 1997; Cheng and Deutscher, 2003; Cameron et al., 2018). PNPase is related in evolution to the exosome assembly of archaea and eukaryotes (Cameron et al., 2018). Although PNPase can catalyze the polymerization of nucleotides, its main cellular function is the 3ʹ-to-5ʹ degradation of RNA (Grunberg-Manago et al., 1955). This reaction, phosphorolysis, requires inorganic phosphate and magnesium ions as cofactors and releases ribonucleoside diphosphates (rNDPs) as products (Nurmohamed et al., 2009).

In bacteria, PNPase is involved in bulk mRNA turnover and the processing of tRNA and rRNA precursors and, in *Listeria monocytogenes* and *Staphylococcus epidermidis*, participates in CRISPR systems (Sesto et al., 2014; Chou-Zheng and Hatoum-Aslan, 2019). Deletion of PNPase is known to reduce virulence and increase sensitivity to stressors (Cameron et al., 2018). In the past decade it has become clear that PNPase also plays a key role in the regulation of gene expression via tight control over the cellular pool of small regulatory RNAs (sRNAs). Between 50 and 200 nt long, these riboregulatory RNAs base pair with other RNAs and influence their stability or translational efficiency (Waters and Storz, 2009; Dendooven and Luisi, 2017). The sRNAs contribute to extensive regulatory networks that mediate control of metabolism, virulence, and other complex processes, and their actions are facilitated by chaperones such as the highly conserved Hfq, a member of the Lsm/Sm protein family (Updegrove et al., 2016). Hfq binds hundreds of small noncoding RNAs and improves their efficacy by aiding base pairing to target mRNAs (Vogel and Luisi, 2011). One long-standing puzzle is how a nascent sRNA gains access to the limited numbers of Hfq that are available in the cell.

Deletion of the PNPase gene from the *E. coli* or *Salmonella* chromosome stabilizes many transcripts (Bernstein et al., 2004; Viegas et al., 2007; Andrade and Arraiano, 2008), as expected, but paradoxically also results in an increased turnover of many sRNAs (De Lay and Gottesman, 2011; Bandyra et al., 2016). The latter in turn results in a loss of efficiency with which the sRNAs can control the expression of targeted genes (De Lay and Gottesman, 2011; Pobre and Arraiano, 2015; Cameron and De Lay, 2016; Dressaire et al., 2018; Cameron et al., 2019). The chaperoning role of PNPase appears nuanced, as it is dependent on growth phase, and not all sRNAs are destabilized by the absence of the enzyme. Moreover, PNPase activity on some mRNAs might also fluctuate between degradation and stabilization, as it was shown that upon ribosomal protein S1 overexpression PNPase protects some transcripts from destruction (Briani et al., 2008). Nonetheless, these findings indicate that PNPase could be a pleiotropic regulator of gene expression, depending on cellular context and information encoded in the sRNA, with additional functions beyond its well-established ribonuclease activities.

It has been demonstrated that the protective mode of PNPase originates in a “RNA carrier” complex it forms with the RNA chaperone Hfq and sRNA (Bandyra et al., 2016). This association with Hfq protects sRNAs from degradation by PNPase in these RNA carrier assemblies, which have been postulated to facilitate downstream action of some regulatory RNAs (Cameron et al., 2019). Among the sRNAs stabilized by PNPase are CyaR, which regulates catabolite repression, quorum sensing, and nitrogen assimilation in *E. coli* (Johansen et al., 2008; Papenfort et al., 2008; De Lay and Gottesman, 2009), and RyhB, which is predicted to regulate more than 50 genes, the majority of which are related to iron homeostasis (Massé and Gottesman, 2002; Massé et al., 2005), and contribute to the pathogenicity of *E. coli* (Zhang et al., 2018). Interestingly, this sRNA regulator itself is regulated by the 3ʹETS^leuZ^, an RNA sponge that titrates out RyhB and RybB, precluding them from acting on their mRNA targets. 3ʹETS^leuZ^ arises from the 3ʹ external transcribed spacer of the glyW-cysT-leuZ pre-tRNA transcript (Lalaouna et al., 2015, 2017), and its activity is regulated via polyadenylation dependent degradation (Sinha et al., 2018). 3ʹETS^leuZ^ was recently shown to interact with several other sRNAs, such as *smpB*, *rpsB*, and DsrA, which suggests that the regulation through RNA sponges might be widespread (Lalaouna et al., 2015, Melamed et al., 2016).

We present results that illuminate how *E. coli* PNPase in conjunction with Hfq can switch between protective and degradative functional modes. Cryoelectron microscopy (cryo-EM) was used to solve structures for *E. coli* PNPase in the apo- and substrate-bound forms and in a ternary complex with sRNA substrate and Hfq (i.e., the RNA carrier complex). In our model of the RNA carrier complex, Hfq cooperates with the KH and S1 structural modules of PNPase to capture the RNA and prevent the 3ʹ end from entering the central channel, thereby safeguarding it from degradation. A degenerate ARN-repeat sequence in the RNA substrate interacts with one of the Hfq RNA-binding surfaces, bridging Hfq and PNPase, and indicating a loose sequence preference for carrier assembly. *In vivo* reporter assays indicate the KH-S1-sRNA interactions in the RNA carrier assembly are important for cellular stability of sRNAs. We also demonstrate how the PNPase-Hfq carrier complex protects sRNAs from degradation by other ribonucleases *in vitro* and at the same time can facilitate handover of the sRNA to its RNA target. These results show how ribonucleoprotein complexes involving RNA chaperones can support RNA-mediated control processes and contribute to their regulatory repertoire.

## Results

### The path of RNA engaged by *E. coli* PNPase in phosphorolytic mode

**Figure 1:**
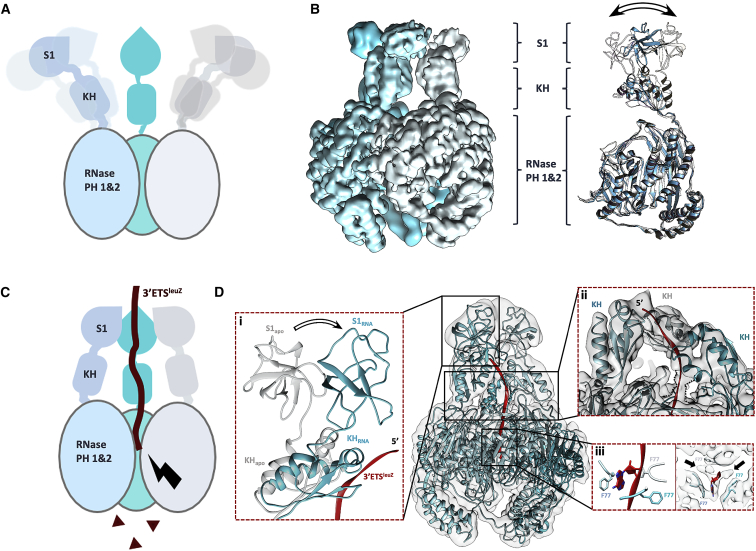
Cryo-EM model of PNPase in apo form and in phosphorolytic mode (A) Schematic overview of a PNPase trimer with a flexible KH-S1 portal. (B) Combined map of PNPase in its apo form after symmetry expansion and masked 3D classification. Symmetry expansion (C3) using Relion resolved the KH and S1 domains and revealed three significantly different states for the KH-S1 modules. Molecular motions of the KH and S1 domain of a PNPase protomer are depicted in the overlayed models on the right and shown in the accompanying videos (Video S1). (C) Schematic of PNPase in phosphorolytic mode. Substrate RNA is bound by the KH and S1 domains and guided toward the core, where nucleoside diphosphates (brown triangles) are liberated processively from the 3ʹ-end via phosphorolysis. (D) Cryo-EM model for PNPase engaged on 3ʹETS^leuZ^ substrate. (i) The KH and S1 domains undergo significant repositioning upon RNA binding, wrapping around the RNA substrate in a more closed conformation (Video S2). (ii) Single-stranded RNA threads toward the core via the KH domains. One of three KH domains seems to contribute to RNA coordination. (iii) Stacking of a base against one of three Phe77 residues marks the entry point into the catalytic PNPase core (left). Extra density was observed for bases stacking against the other Phe77 residues, but these sites are not occupied simultaneously, because of steric restrictions (black arrows). To improve clarity of presentation, the PNPase-3ʹETS^leuZ^ cryo-EM map was low-pass-filtered to 7 Å. The RNA backbones shown in (D) correspond to 4, 7, and 3 nt for (i), (ii), and (iii) respectively. A total of 8 nt were traced in the density.

Structures of *E. coli* apo PNPase have been limited to the catalytic core and parts of the KH domains (Nurmohamed et al., 2009). A full structure has remained elusive, but it has been assumed to have flexibly tethered KH and S1 domains protruding outward from each PNPase protomer. While the catalytic core performs the phosphorolytic action, the KH and S1 domains participate in RNA substrate capture and autoregulation (Wong et al., 2013) and are powerful facilitators of PNPase activity. We investigated full-length PNPase using cryo-EM, which yielded three-dimensional (3D) reconstructions with well-defined density for the RNase PH core at a global map resolution of 3.4 Å and three distinct conformational sub-states for the KH-S1 domains (Figures 1A and 1B; Video S1; Figures S1, S4A, and S4B; Table 1).

**Table 1:** Cryo-EM data collection and refinement statistics for PNPase structures

Structure	PNPase	PNPase-3′ETS^LeuZ^	PNPase-3′ETS^LeuZ^-Hfq
PDB code	7OGL	7OGK	7OGM
EMDB code	12883	12882	12884

Data collection			

Microscope	FEI Titan Krios	FEI Titan Krios	FEI Titan Krios
Voltage (kV)	300	300	300
Detector	Gatan K2	Gatan K2	Gatan K2
Nominal magnification	130,000	130,000	130,000
Pixel size (Å)	1.065	1.065	1.065
Electron dose, per frame (e^–^/Å^2^)	1.40	1.41	1.45
Electron dose, total (e^–^/Å^2^)	53.0	53.6	54.1
Defocus range (μm)	−1/−2.5	−1/−2.5	−1/−2.5
Exposure (s)	12	12	12
Frames	38	38	38
Number of micrographs	1,008	2,741	19,566

Reconstruction			

Software	Relion-3.0.8	Relion-3.0.8/cryoSPARC 2.15	Relion-3.0.8
Number of particles used	58,000	206,803	133,607
Final resolution, FSC_0.143_ (Å)	3.4	3.4	3.7
Map-sharpening B factor (Å^2^)	−76	−110	−123

Model composition			

Non-hydrogen atoms	15,921	16,091	19,918
Protein residues	2,085	2,085	2,454
RNA nucleotides	0	8	50
Molar mass (kDa)	231	253	318

Refinement			

Software	Refmac5/Phenix/Isolde	Refmac5/Phenix/Isolde	Refmac5/Phenix/Isolde
Correlation coefficient, masked	0.85	0.84	0.84
Correlation coefficient, box	0.88	0.86	0.86
FSC_0.5_ (model-map)	3.4	3.6	3.8
Validation (proteins)			
MolProbity score	1.44	1.33	1.7
Clash score, all atoms	3.16	2.23	6.4

Ramachandran plot statistics			

Favored, overall (%)	95.27	95.24	95.73
Allowed, overall (%)	4.49	4.62	3.98
Outlier, overall (%)	0.24	0.14	0.29

RMSDs			

Bond length (Å)	0.0064	0.014	0.0072
Bond angle (°)	1.55	1.55	1.52

Video S1. Morph between conformational sub-states of the KH and S1 domains as seen from the top of PNPase, related to Figure 1 2

We next explored the role of the KH and S1 domains in the degradative processing mode of PNPase. Cryo-EM studies were carried out on complexes formed between PNPase and RNA under conditions that do not support catalysis (Figures 1C and 1D). We used the sponge RNA 3ʹETS^leuZ^, which was observed *in vivo* in pull-downs of PNPase under conditions of induced RyhB expression (Figure S6). In a refined cryo-EM map, at 3.4 Å global resolution (Figure S1B; Table 1), 3ʹETS^leuZ^ threads as a single-stranded RNA over the surface of the S1 and KH domains and through the pore entrance to the central channel (Figure 1Dii). The interaction between the S1 domains and an RNA substrate when PNPase is in phosphorolytic mode is visualized here for the first time (Figure 1Di). The observed path of the RNA along the KH domains is in agreement with the interactions seen in the co-crystal structure of *C. crescentus* PNPase with RNA (Hardwick et al., 2012). In particular, one of the three KH domains appears to be the main contact for the RNA substrate. Even though the local resolution near the KH domains does not allow confident modeling of side chains, there is clear density for the single-stranded RNA backbone (Figure 1Dii). Comparison of the models for PNPase-RNA and apo-PNPase shows that both KH and S1 domains clamp onto the 3ʹETS^leuZ^ substrate (Figures 1A and 1C; Video S2). Although density for RNA is not apparent within the active sites of the enzyme, there is visible density for three bases stacking on the three Phe77 residues at the entrance channel, where the RNA path may branch among three possible routes into the core interior (Figure 1Diii).

Video S2. Morph between apo-PNPase and PNPase-3ʹETSleuZ, related to Figure 1 The KH and S1 domains clamp around the substrate RNA.3

### Cryo-EM model of the PNPase-Hfq-3ʹETS^leuZ^ ternary complex reveals the structural basis for the RNA carrier complex formation

**Figure 2:**
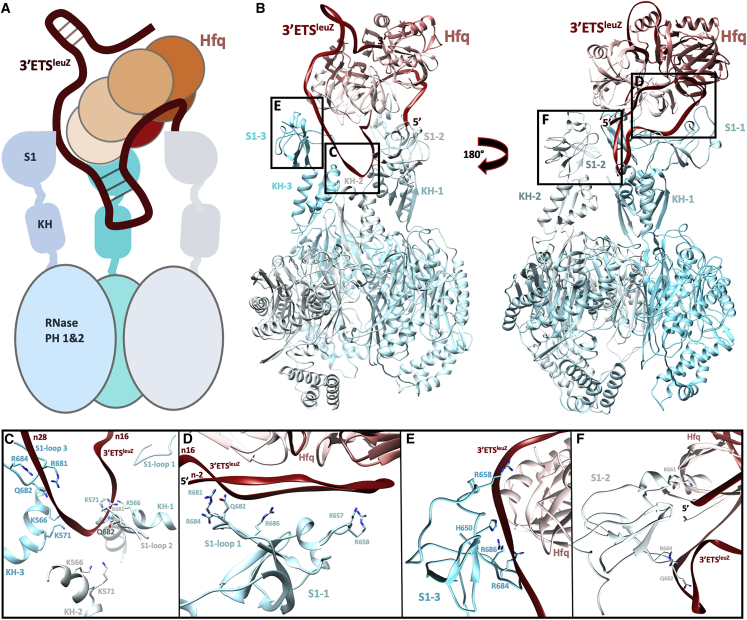
Architecture of the RNA carrier complex (A) Schematic model of the PNPase-Hfq-3ʹETS^leuZ^ RNA carrier complex. (B) Model of the PNPase-Hfq-3ʹETS^leuZ^ RNA carrier assembly. Rerouting of the sRNA, mediated by Hfq, prevents it from being degraded by PNPase. No direct protein-protein contacts between PNPase and Hfq were found; instead, the complex is stabilized by shared RNA interactions, and it is apparent that the KH and S1 domains are crucial for complex formation with Hfq-sRNA. The RNA backbone for 49 residues was traced in the cryo-EM density. (C) All three KH domains (residues K566 and K571) and a basic loop on two S1 domains (S1-loop 2 and S1-loop 3; residues R681, Q682, R684, and R686) coordinate a 3ʹETS^leuZ^ stem-loop on the Hfq distal side. The length of the RNA backbone in this panel corresponds to 12 nt. (D) The same basic loop on a third S1 domain (S1-1), coordinates the 3ʹETS^leuZ^ RNA bound to the Hfq distal side via residues described above and in addition K657 and R658. The length of the RNA backbone in this panel corresponds to 18 nt. (E) S1-3 helps coordinate the sRNA as it threads over the Hfq rim toward the proximal side. The length of the RNA backbone in this panel corresponds to 9 nt. (F) S1-2 appears to bind and coordinate the 5ʹ end of 3ʹETS^leuZ^ in the RNA carrier assembly. The length of the RNA backbone in this panel corresponds to 15 nt.

We next sought to obtain a structure of the PNPase-3ʹETS^leuZ^ complex that operates in conjunction with Hfq in the protective mode. The addition of Hfq to the sample resulted in the formation of a stable and uniform PNPase-Hfq-3ʹETS^leuZ^ ternary complex, which yielded distinct particles on cryo-EM grids (Figures S2A and S2B). We have also explored several other PNPase-Hfq-sRNA carrier complexes and found that despite formation of stable ternary assemblies (Bandyra et al., 2016), no high-resolution reconstructions could be generated, because of conformational heterogeneity. Extensive 3D classification of the images from the PNPase-3ʹETS^leuZ^ complex revealed a series of sub-states and recognizable density for the Hfq chaperone near the KH and S1 domains (Figures S4C and S3). The best class was refined to an overall resolution of 3.8 Å, ranging from 3.2 Å in the PNPase core to 4.4–7.2 Å in the Hfq binding region (Figures S1C, S2C, and S2D; Table 1). At these resolutions, the path for the 3ʹETS^leuZ^ RNA backbone was clearly defined, with interpretable density for the bases in the best regions (Figures 2A and 2B; Figure S2D). Secondary structures were well resolved for all three KH domains and one S1 domain. Refinement of models in the different subclasses reveal likely modes of conformational switching (Figure S4C; Video S3). The two main conformations were each subjected to 3D variation analysis, which identified residual “rocking” and “rotary” modes of molecular motion within each conformational sub-state of the PNPase-Hfq-3ʹETS^leuZ^ assembly (Figures S4C and S4D; Videos S4 and S5).

Video S3. Morph between three conformational sub-states of the PNPase-Hfq-3ʹETSleuZ as resolved by heterogeneous refinement in CryoSparc, related to Figure 2 4

**Figure 3:**
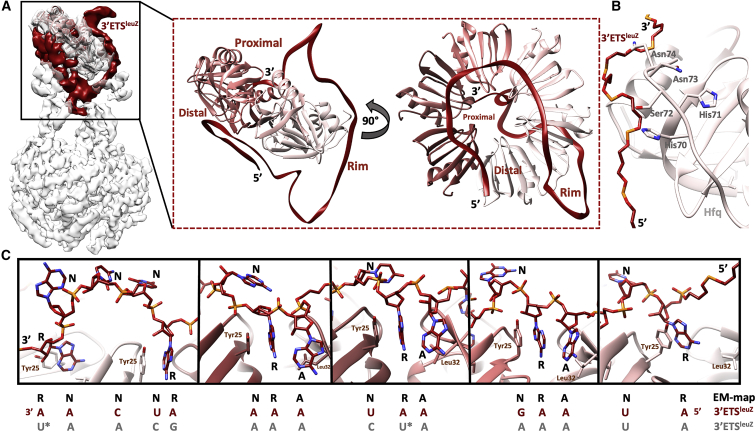
3ʹETS^leuZ^ interactions with Hfq (A) 3ʹETS^leuZ^ binds the Hfq distal side, proximal side, and rim. The parts of 3ʹETS^leuZ^ on the distal side and rim are presented to PNPase. (B) Interpretable density was observed for part of the C-terminal tail of one of the Hfq protomers. Although the local resolution did not allow to model side chains, polar (Ser72, Asn73-74) and charged (His 70-71) residues on the Hfq C-terminal tail could help coordinate the RNA on the Hfq rim-proximal side cross-over region. (C) An incomplete and degenerate ARN repeat motif is bound as a ring-like fold on the Hfq distal side, as observed previously for a perfect 6xARN motif (Link et al., 2009; Pei et al., 2019). All R pockets are occupied, yet only three A pockets are used. In regions where the corresponding base does not occupy the A pocket, the 3ʹETS^leuZ^ backbone is slightly detached from the Hfq distal side and interacts with PNPase S1 domains (left panel and right panel). The resulting -RNARNARNARNRNNNR- motif occurs once in the 3ʹETS^leuZ^ sequence, as annotated in red. An alternative ARN-rich sequence further downstream in the sRNA could be mapped onto the motif (annotated in grey), albeit with two mild violations of the ARN-rule (asterisk). The map in (A) is locally filtered according to the estimated local resolutions and blurred for presentation.

Video S4. “Rocking” motion of the KH-S1-Hfq module in the ternary complex, related to Figure 3 3D variability analysis of the particle images was performed in cryoSPARC (Punjani et al., 2020).5

Video S5. “Rotary” movement of the KH-S1-Hfq module in the ternary complex, related to Figure 3 3D variability analysis of the particle images was performed in cryoSPARC (Punjani et al., 2020).6

It is clear from our cryo-EM data that 3ʹETS^leuZ^ mediates the association of PNPase and Hfq in the RNA carrier assembly and that no direct protein-protein contacts are made. The 3ʹETS^leuZ^ is engaged extensively with Hfq, interacting with the distal and proximal surfaces, and also the circumferential rim (Figures 2A and 2B). This mode of interaction of an sRNA, in which the three RNA-binding surfaces of Hfq are contacted, resembles closely the proposed binding mode of class II sRNAs (Schu et al., 2015), one of the Hfq-dependent sRNA species that include relatively stable sRNAs dependent on the proximal and distal faces of the chaperone.

The RNA in the carrier complex is contacted at multiple sites by the KH and S1 domains of PNPase. A short, exposed hairpin threads into the KH-S1 portal, where it is mainly bound by basic loops extending from two PNPase S1 domains (Figures 2B and 2C, identified as S1-3/S1-loop3 and S1-2/S1-loop2). The same RNA hairpin is in proximity to basic residues on all three KH domains (Figures 2B and 2C) where the RNA folds back, away from the active site. In our model, two of the S1 domains (denoted as S1-2 and S1-3) are involved primarily in coordinating the 5ʹ end of 3ʹETS^leuZ^ (Figures 2B and 2F) and a single-stranded region of the RNA that extends over the Hfq rim (Figures 2B and 2E), respectively. The third S1 domain (labeled S1-1) extensively coordinates an A-rich region of the 3ʹETS^leuZ^ RNA bound to the Hfq distal side (Figures 2B and 2D, S1-1 and S1-loop1) and is the best resolved S1 domain in the cryo-EM map (Figures S2C and S2D). Overall, the same residues on the three KH and S1 domains are involved in RNA binding (Figure 2). Interestingly, in the RNA carrier mode of PNPase the KH and S1 domains adopt an open conformation that more closely resembles the conformation of the apo state rather than the substrate-bound state (Figures 1A, 1C, and 2A).

### An imperfect ARN repeat supports ternary complex formation at the Hfq-PNPase interface

In the proposed binding mode of class II sRNAs, a 3ʹ poly-uridine (poly-U) tail associates with the Hfq proximal side, and a 5′ A-rich region interacts with the Hfq distal side (Schu et al., 2015). Indeed, the 3ʹ end of the 3ʹETS^leuZ^ RNA, marked by a poly-U sequence, is bound to the Hfq proximal face (Figure 3A). Moreover, density resolved for the C-terminal tail of one of the Hfq protomers indicates that the tail interacts with the RNA on the Hfq rim and proximal sides (Figures 3A and 3B). The 3ʹETS^leuZ^ RNA interacts with the distal face of Hfq through a motif observed for other Hfq complexes in Gram-negative bacteria (Link et al., 2009; Pei et al., 2019). This motif, with the consensus pattern A-R-N (where A is adenine, R is purine, and N is any nucleotide), has A and R buried in the distal face, while the N base is exposed (Link et al., 2009). Although the limited local resolution did not allow unambiguous mapping of the 3ʹETS^leuZ^ sequence in the cryo-EM model, we were able to distinguish occupied A and R pockets on the Hfq distal side from empty ones in the cryo-EM reconstruction. Interestingly, not all six “A” pockets on the Hfq distal side are occupied in our cryo-EM map (Figure 3C). Only three complete ARN triplets could be modeled into the density, yielding an interacting sequence pattern 5ʹ RN ARN ARN ARN RNN NR 3ʹ (Figure 3C). This pattern is present in the 3ʹETS^leuZ^ sequence at the 5ʹ end, in agreement with the expected region according to the cryo-EM map (Figure 3C, red sequence; Figure S5D, underlined in red). As the A pocket optimally accommodates only an adenine base (Link et al., 2009; Pei et al., 2019), these violations of the ARN rule at the 3ʹ end of the pattern allow the 3ʹETS^leuZ^ backbone to lift off the Hfq distal face when interacting with the PNPase S1 domains (Figure 3C, left panel). Strikingly, all six R pockets are occupied on the Hfq distal side (Figure 3C). These observations not only confirm for the first time the fold of class II sRNAs when bound to Hfq but also show how degenerate ARN triplets allow specific assembly formation on the Hfq distal side. An alternative mapping of the 3ʹETS^leuZ^ sequence is possible (Figure S5D, underlined in orange), albeit with two mild violations of the ARN rule, as two R pockets on the Hfq distal side would be occupied by uracil bases (Figure 3C, asterisk; Figure S5D). Local resolutions are not sufficient to unambiguously determine which of the two ARN motifs in 3ʹETS^leuZ^ engages the Hfq distal side in the RNA carrier complex.

### Longer class II sRNAs can engage multiple Hfq chaperones for presentation

**Figure 4:**
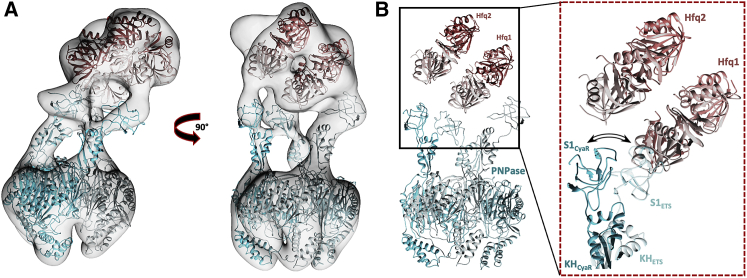
Two Hfq chaperones present CyaR to PNPase (A) A 3D reconstruction reveals two ring-like densities near the KH-S1 domains, corresponding to two Hfq chaperones. (B) A hypothetical model of two Hfq hexamers stacking on top of each other. The orientation of Hfq2 presented here is speculative, and CyaR could not be traced in the cryo-EM map, because of the limited resolution of the reconstructions. One of three KH-S1 pairs is proposed to undergo a significant outward reorganization to coordinate the Hfq2-RNA component (inset).

Other class II sRNAs were also predicted to form RNA carrier assemblies with Hfq and PNPase (De Lay and Gottesman, 2011; Bandyra et al., 2016; Cameron and De Lay, 2016), such as CyaR. Cryo-EM analysis of the PNPase-Hfq-CyaR complex reveals an assembly resembling that of PNPase-Hfq-3ʹETS^leuZ^ (Figure 4). As expected, the KH-S1 portal is conformationally heterogeneous, which poses a resolution limit on the 3D reconstructions (Figures S2E and S2F). Remarkably, the reconstruction shows that the particles contain two stacked Hfq hexamers at the KH-S1 portal (Figure 3B). The large distances between the docked Hfq chaperones and the KH-S1 portal domains suggest that CyaR forms the binding interface between PNPase and Hfq, analogous to the PNPase-Hfq-3ʹETS^leuZ^ assembly. Even though the overall resolution was not sufficient to trace the CyaR backbone, the reconstruction indicates that multiple Hfq chaperones can cooperate to present longer class II sRNAs to the PNPase KH-S1 portal to form RNA carrier assemblies. Correspondingly, PNPase can accommodate different Hfq sub-assemblies via the intrinsic flexibility of its KH and S1 domains. In the PNPase-Hfq-CyaR carrier complex, for example, one of three engaged S1 domains undergoes significant reorganization to coordinate the RNA on the rim of the second Hfq (Hfq2) (Figure 4B). Additional datasets were collected for PNPase-RyhB-Hfq and PNPase-GcvB-Hfq RNA carrier assemblies. Extensive conformational heterogeneity of the complexes and the limited size of the datasets did not allow accurate 3D reconstructions. However, two-dimensional (2D) class averages enabled annotation of the PNPase catalytic core and reveal that the overall quaternary architecture of the RyhB and GcvB RNA carrier complexes is analogous to the CyaR and 3ʹETS^leuZ^ assemblies (Figure S2F). These results further illustrate the potential structural diversity of ribonucleoprotein complexes with which PNPase can cooperate to control sRNA stability and activity in the cell and show how the flexibly tethered KH/S1 portal enables this.

### *In vitro* and *in vivo* validation of interactions in the RNA carrier complex

The model for the PNPase-Hfq-3ʹETS^leuZ^ assembly allowed us to predict and test candidate residues in the PNPase domains that contact the sRNA in the RNA carrier complex. Three PNPase mutants were designed and evaluated for RNA and Hfq complex formation *in vitro*: (1) a double mutant of residues K657A and R658A in the S1 domain implicated in the interaction with RNA on the distal side of Hfq (S1 domain; PNPase S1x2); (2) a double mutant of the KH domain residues K566A and K571A involved in binding the RNA stem loop (KH domain; PNPase KHx2); and (3) a quadruple mutant of S1 domain residues R681A, Q682A, R684A, and R686A on a basic loop predicted to bind 3ʹETS^leuZ^ extensively (S1 domain; PNPase S1x4) (Figure 3C). Three sRNAs were chosen to study the effects of these mutations: the 3ʹETS^leuZ^ captured in our higher resolution cryo-EM structures and two sRNAs representing the two classes of Hfq-binding sRNAs, namely, RyhB (class I) and CyaR (class II), which have been shown previously to form RNA carrier assemblies with PNPase (Johansen et al., 2008; De Lay and Gottesman, 2009; Bandyra et al., 2016).

**Figure 5:**
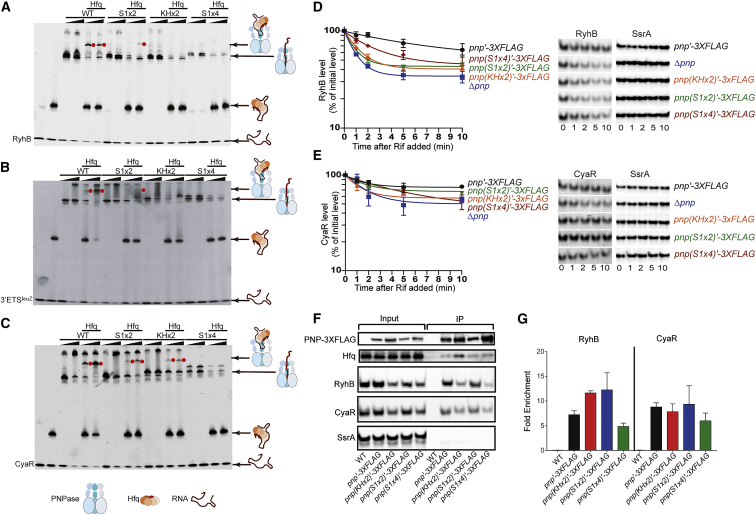
The KH-S1 portal is crucial for PNPase-sRNA-Hfq complex formation (A–C) Electrophoretic mobility shift assays (EMSAs) of wild-type PNPase and KH-S1 mutants with 400 nM RyhB (A), 3ʹETS^leuZ^ (B), and CyaR (C) in the absence and presence of 400 nM Hfq hexamer. Ternary complexes are highlighted with a red dot. Two different PNPase concentrations were used for every PNPase construct (1:1 and 1:3 RNA:PNPase trimer molar ratio), represented by a concentration bar. (D and E) RNA half-life experiments to determine RyhB and CyaR sRNA stabilities in an *E. coli* strain expressing a 3X-FLAG tagged construct of PNPase WT and mutants. RyhB and CyaR signal intensities were quantified using northern blots and normalized to their corresponding loading controls (SsrA). sRNA decay curves were generated by fitting the normalized signal intensities for each time point. Points and error bars in the curves represent the means and the standard errors (SEM) of at least three independent experiments. Northern blots for RyhB and CyaR half-life measurements corresponding to RNA stability curves are shown and values tabulated in Table S2. (F and G) Cell extracts prepared from late exponential phase cultures of *E. coli* strains expressing WT PNPase, or FLAG-tagged PNPase WT and mutants were used to assess coprecipitation of sRNAs, which were analyzed using northern blot. (G) Fold enrichment of a given RNA upon immunoprecipitation was determined by first calculating the signal intensity per microgram of RNA for the input and the elution from the northern blots in (F). The normalized elution signal was then divided by the input signal. An untagged wild-type strain (WT) was used as a control for data presented in (F) and (G). S1x2: PNPase K657A, R658A; KHx2: PNPase K566A, K571A; S1x4: PNPase R681A, Q682A, R684A, R686A.

All purified PNPase mutants were able to degrade RNA substrates but exhibited lower levels of activity compared with wild-type enzyme (Figure S5), in agreement with the hypothesis that the KH and S1 domains support substrate capture. However, in the presence of Hfq, none of the PNPase mutants nor the wild-type enzyme can degrade the sRNAs, suggesting Hfq masks the RNA from the PNPase catalytic core (Figure S5). Efficient PNPase-sRNA-Hfq ternary complex formation could not be observed for any of the PNPase mutants and any of the three RNAs tested, although PNPase S1x2 and KHx2 in some cases still showed a reduced affinity for Hfq-bound sRNA (Figures 5A–5C). The PNPase S1x4 mutant is not able to form a ternary complex with Hfq and sRNA for any of the tested sRNAs. These results are in agreement with the proposed model for the RNA carrier mode of PNPase, which predicts that the basic loop in the S1 domain is paramount in formation of the assembly (Figures 3B and 3C).

To determine the contribution of the KH/S1 domains in the context of the RNA carrier assembly to sRNA metabolism *in vivo*, we first examined the impact of the PNPase mutants on CyaR and RyhB stability after inhibition of transcription initiation by rifampicin treatment. As shown in Figure 5D and Table S2, all three sets of substitutions resulted in increased turnover of RyhB. CyaR stability significantly decreased in strains expressing PNPase KHx2 and S1x4, whereas expression of PNPase S1x2 caused a modest reduction in the stability of this sRNA compared with the PNPase-3xFLAG (Figure 5E). We also observed a modest effect of PNPase mutations on 3ʹETS^leuZ^ stability (Table S2). Next, we compared the ability of the wild-type and mutant forms of the PNPase constructs to interact with RyhB and CyaR via PNPase co-immunoprecipitation assays. These experiments show that the PNPase S1x4 mutant was defective in binding CyaR and RyhB, whereas the PNPase S1x2 and KHx2 pulled down a similar amount of these RNAs as the PNPase-3xFLAG (Figures 5F and 5G). Altogether, these *in vivo* findings are consistent with the *in vitro* results indicating that a positively charged loop within the S1 domain formed by R681, Q682, R684, and R686 is important for sRNA interaction and stabilization *in vivo*, probably because of its indispensability in RNA carrier complex formation, and that positively charged residues in the KH domain have a significant, but less substantial role in the RNA carrier assembly.

### Downstream effector roles for PNPase-Hfq RNA carrier assemblies

We next explored if and how the stabilizing complexes facilitate sRNA function. As PNPase was shown to increase stability of some sRNAs in the cell, and was unable to degrade the sRNA in the RNA carrier mode (Figure S5), we tested if the RNA carrier assembly can protect sRNAs from other cellular ribonucleases.

**Figure 6:**
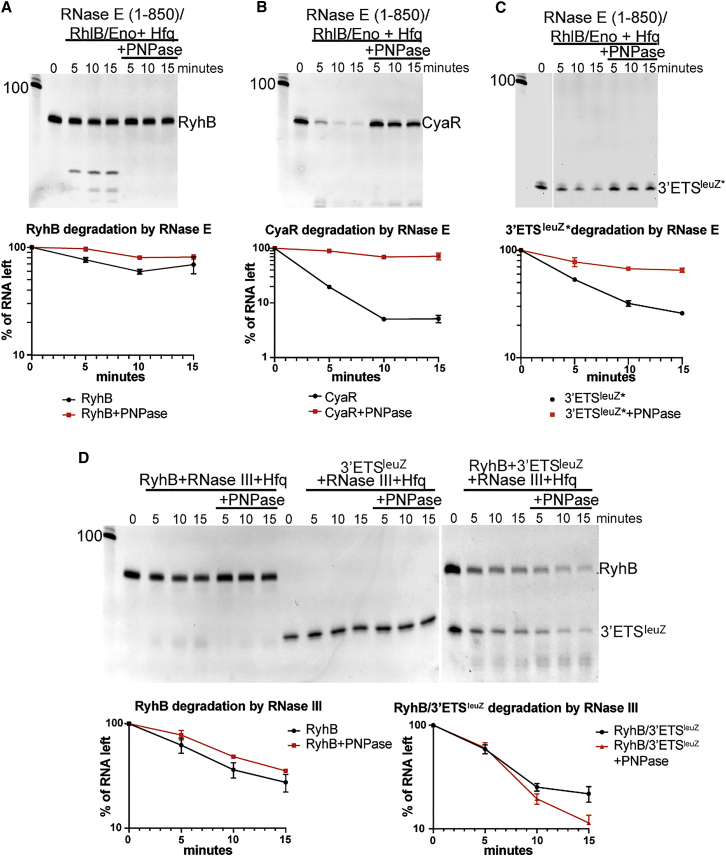
RNA carrier assemblies protect sRNAs from RNase E and facilitate target pairing (A–C) Degradation of RyhB (A), CyaR (B), and 3ʹETS^leuZ∗^ (C) in the absence and presence of PNPase by RNase E. 3ʹETS^leuZ∗^ is the 5ʹ monophosphorylated processed form of 3ʹETS^leuZ^. (D) Degradation of RyhB, 3ʹETS^leuZ^, and the RyhB-3ʹETS^leuZ^ complex by RNase III. For all experiments, 200 nM RNA was incubated with 200 nM Hfq hexamer and 7.5 nM tetramer of RNase E (1–850)/RhlB/Enolase or 0.01 U (RNAs alone) or 0.005 U (RyhB and 3ʹETS^leuZ^ together) RNase III, in the presence or absence of 200 nM PNPase trimer. Reaction times, in minutes, are given in the lanes above the gels. Data are represented as mean ± SD.

First, we investigated RNase E cleavage of RyhB and CyaR, which have both been shown to be cleaved by this enzyme (Massé et al., 2003; Kim and Lee, 2020). We used a truncated version of RNase E (residues 1–850) that lacks the PNPase binding site and forms a truncated RNase E sub-assembly together with the RNA helicase RhlB and the glycolytic enzyme enolase (RNase E [1–850]/RhlB/Eno; truncated degradosome). As shown in Figure 6, both sRNAs are more resistant to RNase E cleavage when wrapped in an RNA carrier assembly. RyhB is not cleaved efficiently by RNase E in the presence of Hfq (Figures 6A and 6B), and the formation of the RNA carrier complex increases its stability only modestly. For CyaR, however, the presence of PNPase has a substantial impact on its stability against RNase E (Figure 6B). Notably, RNase E cleaves Hfq-associated CyaR rapidly, suggesting that some sRNAs remain vulnerable to ribonucleases when associated with the chaperone. However, in the PNPase-Hfq-CyaR complex, the sRNA is protected from RNase E attack *in vitro* (Figure 6B). We have also tested the stability of the 53-nt-long processed form of 3ʹETS^leuZ^ against RNase E cleavage (Lalaouna et al., 2017; 3ʹETS^leuZ∗^). This processed form of 3ʹETS^leuZ^ has a monophosphorylated 5ʹ end, making it a suitable substrate for RNase E. We confirmed that the processed 5ʹP-3ʹETS^leuZ∗^ can form a PNPase-Hfq-3ʹETS^leuZ^^∗^ complex (Figure S6E). Next, we investigated the stability of this RNA against RNase E cleavage. 5′P-3ʹETS^leuZ∗^ is degraded by RNase E in the presence of Hfq, but formation of the carrier PNPase-Hfq-3ʹETS^leuZ∗^ complex increases the stability of this sRNA against RNase E (Figure 6C).

We noted that the seed region of 3ʹETS^leuZ^ that matches RyhB is partially exposed on the rim of Hfq in the ternary complex with PNPase, directly following the ARN-repeat motif (Figure S5D). To explore this potential interaction platform, we tested RyhB degradation in the presence of the 3ʹETS^leuZ^ sponge. The RyhB-3ʹETS^leuZ^ pair of RNAs was not degraded by RNase E, which acts on RyhB only in the presence of the processed, 5ʹ monophosphorylated 3ʹETS^leuZ∗^ (results not shown). However, the base-pairing region between these RNAs spans 19 nt (Lalaouna et al., 2015). This makes the RyhB-3′ETS^leuZ^ duplex a good substrate for the RNA duplex-cleaving ribonuclease RNase III, which can be used as a structural probe for RNA pairing. Notably, RNase III cleaves the RyhB-3ʹETS^leuZ^ pair very efficiently (Figure 6D), and the presence of PNPase increased the rate of RNA duplex digestion. The presence of PNPase in addition to Hfq results in a modest increase (8%) in RyhB stability in absence of 3'ETS^leuZ^, compared to the reaction with only Hfq. In contrast, RyhB-3ʹETS^leuZ^ degradation in the presence of PNPase was approximately 10% faster than when only Hfq was present in the reaction (Figure 6D). Although modest, these results suggest that the PNPase-sRNA-Hfq ternary complex may facilitate sRNA pairing to targets.

In summary, these results suggest that PNPase not only protects Hfq-bound sRNA species from degradation by other ribonucleases but could also increase the efficiency of degradation in the presence of a base-pairing RNA partner. As such, PNPase may boost the facilitating role of Hfq to pair some sRNAs with their corresponding target RNAs *in vivo*, although this warrants further investigation.

## Discussion

**Figure 7:**
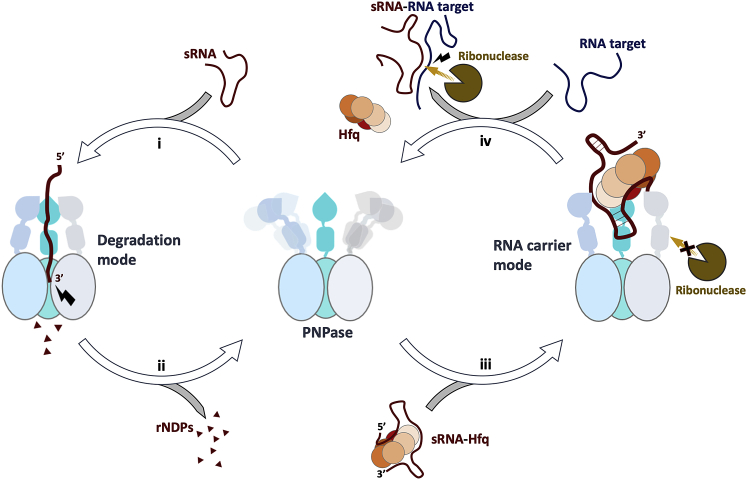
Schematic overview of the degradative and protective modes of PNPase activity PNPase (middle) can capture an RNA substrate via its flexibly tethered KH-S1 portal for degradation in the core (i), releasing nucleoside diphosphates (ii). When the sRNA is presented by Hfq, PNPase switches to its RNA carrier mode, lending its KH-S1 RNA recognition module as a binding platform (iii). When wrapped in an RNA carrier assembly, the sRNA can be protected against other ribonucleases, such as RNase E. If an RNA target engages the RNA carrier complex, the sRNA is released and can pair with the target, which results in dissociation of the PNPase-Hfq-sRNA ternary complex (iv). Ribonucleases, including PNPase, can now cleave the RNA target.

PNPase has been studied for more than five decades as a potent enzyme for degrading RNA, but even the widely investigated exonucleolytic activity of PNPase is not thoroughly understood. As a highly conserved enzyme, PNPase activity is indispensable for many crucial physiological processes, including RNA degradation and processing, CRISPR-based bacterial immunity systems, and homeostasis of human mitochondria. Our cryo-EM models for *E. coli* apo-PNPase and PNPase engaged on an RNA substrate explain how the enzyme captures and degrades RNA. The structure of *E. coli* PNPase in apo form reveals for the first time the wide conformational landscape of the KH and S1 RNA recognition modules (Figure 7). The structure of the substrate-bound enzyme in the phosphorolytic mode indicates how the flexibility of the S1 and KH domains facilitates substrate capture and how these domains clamp down on intercepted single-stranded RNA (Video S2). These observations support the hypothesis that the loose “beads on a string-like” organization of the KH and S1 domains enable PNPase to efficiently capture various RNA targets for degradation.

Only recently has PNPase been recognized for other roles beyond RNA degradation. The paradoxical observation that sRNAs have reduced stability in PNPase-knockout strains support the hypothesis of additional roles in riboregulation (De Lay and Gottesman, 2011; Pobre and Arraiano, 2015; Bandyra et al., 2016; Cameron and De Lay, 2016). For Hfq-dependent sRNAs, such as RyhB, GcvB, CyaR, and MgrR, PNPase was shown to degrade the molecules in the absence of Hfq but to stabilize them when presented by the RNA chaperone (Andrade et al., 2012; Bandyra et al., 2016; Cameron and De Lay, 2016). Our structural data reveal for the first time how Hfq and the PNPase KH-S1 portal cooperate to coordinate an sRNA and direct it away from the PNPase catalytic core (Figure 7). The specificity of the carrier complex depends on the RNA that must meet the combined requirements to interact with Hfq and simultaneously be appropriately presented at points to permit interaction with the PNPase KH and S1 domains. Where the PNPase-3′ETS^leuZ^-Hfq structure serves as a model for the RNA carrier complex, the CyaR, RyhB, and GcvB carrier complexes were shown here to adopt analogous quaternary structures (Figure 4; Figures S2B and S2F). All three KH domains and all three S1 domains bind 3ʹETS^leuZ^, which is engaged on Hfq. *In vitro* binding assays and *in vivo* stability assays confirm that a series of basic residues on the KH and S1 domains are key for RNA carrier complex formation. Interestingly, Hfq presents 3ʹETS^leuZ^ so that only the PNPase RNA recognition module (KH-S1) is engaged. The capacity of PNPase to form ribonucleoprotein assemblies or bind allosteric ligands reflects on the biological importance of modulating the enzyme (Gatewood and Jones, 2010; Nurmohamed et al., 2011; Tuckerman et al., 2011; Stone et al., 2017). In *Deinococcus radiodurans*, for example, the activity of PNPase can be re-programmed through assembly with a noncoding RNA Y and the Rsr protein, which is an ortholog of the eukaryotic Ro protein proposed to act in quality control mechanisms (Chen et al., 2013). Such ribonucleoprotein assembly participates in degradation of structured RNAs, a function that is not displayed by PNPase alone. A similar complex may exist in *Salmonella enterica* serovar Typhimurium (Sim and Wolin, 2018). Moreover, PNPase is not the only exoribonuclease implicated in RNA protection, as RNase II was also shown to increase stability of *rpsO* transcripts in *E. coli* (Hajnsdorf et al., 1994), emphasizing the functional versatility of these enzymes.

The RNA carrier mode of PNPase could be conserved beyond bacteria. In humans, PNPase (hPNPase) localizes between the mitochondrial inner and outer membranes (Chen et al., 2006). Many roles have been reported for hPNPase, including import of 5S rRNA and RNA components of RNase MRP and RNase P to the mitochondrial matrix (Wang et al., 2010). How hPNPase mediates RNA transport across the mitochondrial intermembrane space (IMS) while not cleaving the RNA remains to be answered, but the KH-S1 portal is likely to play a crucial role. As such, there might be similarities with the way *E. coli* PNPase lends its KH and S1 domains to other effector molecules, such as sRNA-Hfq pairs, mirroring in mammalian cells the complex relationships between PNPase and RNA levels observed in bacteria.

The structures above reveal cooperation between PNPase S1 domain and Hfq to interact with RNA. In *E. coli*, the S1 protein of the ribosomal small subunit has a string of four S1 RNA binding modules that promote accommodation into the decoding channel of the 30S subunit (Duval et al., 2013), and these might form similar interactions with transcripts regulated and bound by Hfq. A recent study on 100S hibernating ribosomes revealed how one of the S1 domains interacts with the anti-Shine-Dalgarno sequence of the 16S rRNA (Beckert et al., 2018). This interaction pattern is similar to the interactions observed for the PNPase-S1 domain (S1-1, Figure 2) and 3ʹETS^leuZ^, as presented by Hfq. Therefore, this model might explain how the Hfq distal side and ribosomal S1 domains could act together on certain transcripts to help determine their fate. In this mode, the proximal face of Hfq would be exposed to recruit sRNAs that can remodel the translation machinery to either support translation or to trigger degradation by ribonucleases such as RNase E.

The results presented here elucidate a novel cellular function for bacterial PNPase. The enzyme does not solely degrade or process RNA substrates, but its KH-S1 portal can form a complex interaction platform for sRNAs and their RNA targets, as well as associated effector proteins. In *E. coli,* repurposing of PNPase into this RNA carrier mode is triggered by Hfq-mediated presentation of a sRNA with mild sequence specificity. The RNA carrier assembly is likely to accelerate duplex formation between matching RNAs or participate in the handover of the RNA duplex from Hfq to a ribonuclease. Given the role of PNPase in sRNA-mediated regulation, many of the phenotypes associated with loss of PNPase are potentially explained by its dual role in sRNA degradation and stability, contributing a pivotal node in post-transcriptional regulation networks. These findings significantly broaden our understanding of the regulatory repertoire of PNPase, as well as the scope of the Hfq interactome and regulatory complexes it may form.

### Limitations of the study

The conformational heterogeneity of the cryo-EM specimens places limitations on interpreting the details of the interactions, and structural analysis of class I sRNAs has been elusive. These limitations might be overcome with improvements to preparing cryo-EM specimens and advances in computational approaches to model a continuum of conformational states. The *in vivo* validation of the proposed complexes and analysis of their biological function has been challenging because of the transient nature and potentially complex stoichiometric composition of the assemblies. Addressing this will require *in vivo* approaches to trap and stabilize complexes *in situ* for isolation and characterization.

## STAR★Methods

### Key resources table

**Table:** 

REAGENT or RESOURCE	SOURCE	IDENTIFIER
**Antibodies**		

Rat monoclonal anti-FLAG	Agilent Technologies	Cat#200474; Lot#0006038691
Rabbit monoclonal anti-Hfq	Susan Gottesman, NCI	N/A

**Bacterial and virus strains**		

KR10000 MG1655 *rph*^*+*^	Donald Court, NCI	N/A
*Escherichia coli* BL21 (DE3)	Thermofisher	C600003
CR201 *cya::kan ccdB*	C. Ranquet, Université Joseph Fourier	CR201
NRD999 MG1655 *rph*^*+*^ Δ*pnp::cat*	Bandyra et al., 2016	NRD999
NRD1243 MG1655 *rph*^*+*^*pnp’-3xFLAG*	Cameron and De Lay, 2016	NRD1243
NRD1369 MG1655 *rph*^*+*^ Δ*pnp*	Cameron and De Lay, 2016	NRD1369
NRD1611 MG1655 *rph*^*+*^*pnpΔ566-71::ccdB kan::3xFLAG*	This manuscript	NRD1611
NRD1612 MG1655 *rph*^*+*^*pnpΔ657-8::ccdB kan::3xFLAG*	This manuscript	NRD1612
NRD1613 MG1655 *rph*^*+*^*pnpΔ681-6::ccdB kan::3xFLAG*	This manuscript	NRD1613
NRD1614 MG1655 *rph*^*+*^*pnp(2x2)’-3xFLAG*	This manuscript	NRD1614
NRD1615 MG1655 *rph*^*+*^*pnp(2x1)’-3xFLAG*	This manuscript	NRD1615
NRD1617 BL21(DE3) Δ*pnp::cat*	This manuscript	NRD1617
NRD1622 MG1655 *rph*^*+*^*pnp(4x)’-3xFLAG*	This manuscript	NRD1622
NRD1137 MG1655 *rph*^*+*^*Δpnp::cat rne-131 zce-726:*Tn*10*	Cameron et al., 2019	NRD1137

**Chemicals, peptides, and recombinant proteins**		

Anti-FLAG M2 Affinity Gel	Sigma-Aldrich	Cat#A2220; Lot#SLBN7830V
3X FLAG peptide	ApexBio	Cat# A6001
2,2′-Bipyridyl	Sigma-Aldrich	Cat#D216305
3′,5′-cyclic AMP.Na (cAMP)	Chem-Impex Int’l Inc.	Cat#00008
Phusion High-Fidelity DNA Polymerase	Thermo Fisher Scientific	F530S
T4 DNA ligase	New England Biolabs	M0202
RNaseOUT Recombinant Ribonuclease Inhibitor	Thermo Fisher Scientific	10777019
TURBO DNase (2 U/μL)	Thermo Fisher Scientific	AM2238
ATP	Merck/Sigma-Aldrich	A2383
UTP	Merck/ Sigma-Aldrich	U6625
GTP	Merck/ Sigma-Aldrich	G8877
CTP	Merck/ Sigma-Aldrich	C1506
CHAPSO	Merck/ Sigma-Aldrich	C3649
Restriction enzymes	New England Biolabs	R0189, R0193
Alkaline Phosphatase	New England Biolabs	M0290
SYBR Gold	Thermo Fisher Scientific	S11494
ShortCut® RNase III	New England Biolabs	M0245

**Deposited data**		

Model of apo-PNPase	This manuscript	PDB ID 7OGL; EMD-12883
Model of PNPase with 3′ETS	This manuscript	PDB ID 7OGK; EMD-12882
Model of PNPase with Hfq and 3′ETS	This manuscript	PDB ID 7OGM; EMD-12884

**Oligonucleotides**		

Primers for PNPase 2x1, PNPase 2x2, and PNPase 4x, see Table S1	This manuscript	N/A

**Recombinant DNA**		

pKD46 Amp^R^, RepA101(Ts), λ γ, β, and *exo* expressed from an *araBAD* promoter	Datsenko and Wanner, 2000	pKD46
pTC352 Amp^R^; *P*_*lac*_*araB-5′UTR pnp’-3xFLAG lacI*^*q*^	Cameron et al., 2019	pTC352
pTC354 Amp^R^; *P*_*lac*_*araB-5′UTR pnp(S438A)’-3xFLAG lacI*^*q*^	Cameron et al., 2019	pTC354
pTC396 Amp^R^; *P*_*lac*_*lacI*^*q*^	Cameron et al., 2019	pTC396
pET_Duet_pnpwt	Bandyra et al., 2016	pET_Duet_pnpwt
pET_Duet_pnpS1x2	This manuscript	pET_Duet_pnpS1x2
pET_Duet_pnpKHx2	This manuscript	pET_Duet_pnpKHx2
pET_Duet_pnpS1x4	This manuscript	pET_Duet_pnpS1x4

**Software and Algorithms**		

Relion 3.0	Scheres, 2012; Zivanov et al., 2018	N/A
cryoSPARC	Punjani et al., 2017	N/A
MODELER	Fiser and Šali, 2003	N/A
UCSF Chimera	Pettersen et al., 2004	N/A
CCPEM 1.4.1	Murshudov et al., 2011; Burnley et al., 2017	N/A
COOT	Emsley et al., 2010	N/A
ISOLDE	Croll, 2018	N/A
PHENIX 1.18	Afonine et al., 2018	N/A
MOLPROBITY	Chen et al., 2010	N/A

**Other**		

UltrAuFoil® R 2/2 on Au 200 mesh grids	Quantifoil	N1-A16nAu20-01

### Resource availability

#### Lead contact

Further information and requests for resources and reagents should be directed to and will be fulfilled by the Lead Contact, Ben F. Luisi (bfl20@cam.ac.uk).

#### Materials availability

Plasmids and strains created in this study are available from the Lead Contact upon request.

#### Data and code availability

Cryo-EM maps and models generated in this study are available at EMBD (accession codes PDB ID 7OGK, EMD-12882; PDB ID 7OGL, EMD-12883; PDB ID 7OGM, EMD-12884)

### Experimental model and subject details

All strains used in this study are derivatives of *E. coli* K12 strain MG1655 (RRID:Addgene_61440) or BL21DE3. Depending on the experiment, the strains were grown at 37°C in LB medium (MG1655) or 2xYT medium (BL21) to early or late exponential phase.

### Method details

#### Bacterial strains and plasmids

*E. coli* strains and plasmids used in this study are listed in Table S3. Primers and probes used in this study are listed in Table S1. Oligonucleotides were purchased from Integrated DNA Technologies, Inc. or Sigma-Aldrich Co., LLC.

Strains NRD1611, NRD1612, and NRD1613 were generated as follows. A portion of the *pnp* gene in strain NRD1243 was replaced with a PCR product containing a cassette encoding the kanamycin resistance and *ccdB* toxin genes by lamba Red recombinase-mediated allele replacement. The PCR products used to generate NRD1611, NRD1612, and NRD1613 were amplified via PCR from CR201 genomic DNA using primers pnpD566-71KO For and Rev, pnpD657-8KO For and Rev, and pnpD681-6KO For and Rev, respectively. Successful recombinants were selected using LB plates containing arabinose (0.2%), which causes expression of the CcdB toxin, and validated by PCR using primers pnp For and Rev.

Strains NRD1614, NRD1615, and NRD1622 were created using lambda Red recombinase-mediated allele replacement to exchange the *ccdB kan* cassette located within *pnp* with sequence in the DNA oligos pnpK566K571, pnpK657R658, and pnpRQRR681-6, which contain mutations in portions of *pnp* resulting in the K566A K571A, K657A R658A, and R681A Q682A R684A R686A substitutions, respectively.

Strain NRD1617 was created by lambda Red recombinase-mediated gene replacement exchanging *pnp* with a PCR product containing a chloramphenicol resistance cassette that was amplified from NRD1137 genomic DNA using pnpfar For and Rev primers. Successful recombinants were validated by PCR and sequencing using primers pnpmidFor3 and pnp Rev.

#### PNPase cloning

Mutants of the PNPase gene for *in vitro* studies were prepared by two successive PCR reactions. In the first step, two fragments were PCR-amplified using the wild-type PNPase gene as a template: one using the forward primer PNPaseNcoFor and a reverse primer introducing the mutation (primers PNPaseS1x2Rev or PNPaseKHx2Rev or PNPaseS1x4Rev) (Table S1); the other using a reverse primer PNPaseNotRev and a forward primer introducing the mutation (primers PNPaseS1x2For or PNPaseKHx2For or PNPaseS1x4For) (Table S1). PCR products were resolved on 1% low melting point agarose gel (Sigma), the bands of interest were excised and after melting the matrix at 70°C, they were mixed in one PCR reaction which amplified the entire PNPase gene, with mutations, using primers PNPaseNcoFor and PNPaseNotRev. The product of the last PCR was digested with NcoI and NotI (NEB), resolved on a low melting point agarose gel, and the gel band was directly ligated with T4 ligase (NEB) into a pET duet plasmid, which had been digested with the same restriction enzymes and dephosphorylated with CIP (NEB) according to the manufacturer’s instructions.

#### Protein and RNA purification

*Escherichia coli* PNPase and Hfq were prepared using the protocols described earlier (Bandyra et al., 2016). For expression of PNPase mutants BL21(DE3) Δ*pnp E. coli* strain (NRD1617) was used.

For Hfq preparations, pEH-10-(hfq) was transformed into BL21(DE3) cells. 2 × YT media (Formedium) supplemented with 100 μg/ml carbenicillin was inoculated with starter culture and grown at 37°C. The cultures were induced with 1 mM IPTG at OD_600_ 0.45. 3 h after induction, cells were harvested by centrifugation, resuspended in lysis buffer (50 mM Tris pH 8, 1.5 M NaCl, 250 mM MgCl_2_, 1 mM EDTA, 1 protease inhibitor cocktail tablet (Roche)) and flash frozen in liquid nitrogen. Upon thawing the cells were passed thrice through an EmulsiFlex-05 (Avestin) cell disruptor and the lysate was clarified with centrifugation. Subsequently, the lysate was incubated at 85°C for 20 mins and centrifuged again. Ammonium sulfate was added to the supernatant up to 1.5 M followed by another centrifugation step. The final supernatant was loaded onto a HiTrap Butyl–Sepharose column (GE Healthcare) equilibrated with HfqA buffer (50 mM Tris pH 8.0, 1.5 M NaCl, 1.5 M (NH_4_)_2_SO_4_). Proteins were eluted with an isocratic gradient of HfqB buffer (50 mM Tris pH 8.0) and aliquots of fractionated elutant analyzed by SDS–PAGE. Fractions enriched with Hfq were pooled, concentrated and loaded onto a Sephadex 75 gel filtration column (GE Healthcare) equilibrated with HfqC buffer (50 mM Tris pH 8.0, 100 mM NaCl, 100 mM KCl and 1 protease inhibitor cocktail tablet (Roche)). Fractions were analyzed by SDS–PAGE and the concentration of clean Hfq fractions was determined spectroscopically using a NanoDrop ND-1000 spectrophotometer (Thermo Scientific) and a λ_280nm_ extinction coefficient of 4470 M^-1^cm^-1^ (per Hfq protomer) before the samples were flash frozen and stored at −80°C.

For the PNPase procedure, pET_Duet_pnpwt was transformed into BL21(DE3) (wild-type PNPase) or BL21(DE3) Δ*pnp* (NRD1617) (PNPase mutants). 2 × YT media (Formedium) supplemented with 100 μg/ml carbenicillin was inoculated with precultures and grown at 37°C. The cultures were induced with 0.5 mM IPTG at OD_600_ 0.45 and the temperature was reduced to 25°C. 3-4 h after induction cells were harvested by centrifugation (∼4500 g, 20 min), resuspended in lysis buffer (20 mM Tris pH 8, 150 M NaCl, 150 mM KCl, 5 mM MgCl_2_, 1 mM EDTA, 1 protease inhibitor cocktail tablet (Roche)) and flash frozen in liquid nitrogen.

Upon thawing the cells were passed thrice through an EmulsiFlex-05 cell disruptor (Avestin) at pressure near 1000 bar and clarified by centrifugation (37000 x g, 30 minutes, 5°C). PNPase was precipitated from the lysate with ammonium sulfate (51.3% saturation) at 4°C. The sample was centrifuged and the pellet resuspended in Q Buffer A’ (20 mM Tris-HCl pH 8.5, 0.5 mM TCEP, 10% v/v glycerol, EDTA-free protease inhibitor cocktail (Roche)), loaded on 5 mL HiTrap Q column (GE Healthcare) equilibrated in Q buffer A (20 mM Tris-HCl pH 8.5, 30 mM NaCl, 0.5 mM TCEP, 10% v/v glycerol) and eluted with a 0%–60% gradient of Q Buffer B (Q Buffer A with 1 M NaCl). Fractions from the Q column were evaluated by SDS-PAGE and those containing PNPase were pooled and supplemented with 1mM MgCl_2_, 45 mM Na phosphate pH 7.9, 0.9 M (NH_4_)_2_SO_4_ and 1 mM TCEP and the protein solution was loaded on a 5 mL HiTrap Butyl-Sepharose column (GE Healthcare) equilibrated with BS Buffer A (50 mM Tris-HCl pH 7.5, 1 M (NH_4_)_2_SO_4_, 0.5 mM TCEP) and eluted with a 0%–66% gradient of BS Buffer B (50 mM Tris-HCl pH 7.5). Fractions containing PNPase were pooled, concentrated and loaded on a Superdex 200 10/300 GL gel filtration column (GE Healthcare) equilibrated with a buffer composed of 20 mM Tris-HCl pH 8.0, 150 mM NaCl, 5 mM MgCl_2_, 0.5 mM TCEP, 10% (v/v) Glycerol. Concentration of purified protein was determined spectroscopically using a NanoDrop ND-1000 spectrophotometer (Thermo Scientific) and a λ_280nm_ extinction coefficient of 30370 M^-1^cm^-1^ (per protomer) and it was flash frozen in liquid nitrogen and stored at −80°C. All peak fractions were analyzed by SDS-PAGE.

#### RNA *in vitro* transcription and purification

All RNAs used in this study were prepared by *in vitro* transcription (IVT) using standard protocol. As an IVT template PCR products were used obtained in the reaction with Phire Hotstart II polymerase (Thermo Fisher) according to manufacturer’s instructions (RyhB), or hybridized DNA oligonucleotides (CyaR, 3ʹETS^leuZ^; Sigma). The sequences of used primers and DNA oligonucleotides are summarized in Table S1. RNA was separated on 6% polyacrylamide denaturing gel (National Diagnostics), electroeluted in TBE (Whatman Elutrap) and cleaned up using PureLinkTM RNA Microscale Kit (Invitrogen).

#### RNA binding and degradation assays

Degradation assays were performed and complexes for native gels were assembled as previously described (Bandyra et al., 2016). For the PNPase degradation assays (Figure S5) 0.2 μM RNA was annealed for 2 minutes at 50°C and mixed with Hfq, if appropriate, in degradation buffer (20 mM Tris, pH 7.5, 100 mM NaCl, 1 mM MgCl_2_, 1 mM DTT, 2 mM sodium phosphate). Degradation was started by addition of wild-type or mutant PNPase at 37°C. Samples taken from the reaction were quenched at designated time points by adding and equal volume of 0.5 mg/mL proteinase K, diluted in a buffer containing 100 mM Tris-HCl pH 7.5, 150 mM NaCl, 12.5 mM EDTA and 1% w/v SDS and incubated at 50°C for 20 min. RNA contents corresponding to each time point were run on a 8% polyacrylamide, 7.5M urea gel (National Diagnostics) in 1xTBE and visualized using Sybr Gold (Invitrogen). Quantification was performed with GeneTools (Syngene).

For the truncated degradosome degradation assays (Figure 6) the same procedure was used but samples were prepared in buffer consisting of 25 mM Tris-HCl pH 7.5, 50 mM NaCl, 50 mM KCl, 10 mM MgCl_2_, 1 mM DTT, 0.5 U/μL RNase OUT (Invitrogen). RNase III assays were performed according to manufacturer’s instruction (ShortCut® RNase III; NEB).

For binding assays, 0.4 μM RNA was mixed with indicated proteins in binding buffer (20 mM Tris, pH 7.5, 100 mM NaCl, 1 mM MgCl_2_, 1 mM DTT) and incubated at 30°C for 20 minutes. Subsequently, samples were transferred to ice, supplemented with 5 μL of 50% glycerol in binding buffer and immediately loaded on the PAA gel supplemented with 10% glycerol and run in tris-glycine buffer pH 8.5.

#### Immunoprecipitation of PNPase

PNPase was immunoprecipitated as previously described (Cameron et al., 2019). After cultures of each strain were grown at 37°C in LB medium to late exponential phase (OD_600_ of ∼1.0), RyhB expression was induced for 15 min by the addition of bipyridyl (250 μM). Cells were then harvested by centrifugation, washed twice with a TBS solution (50 mM Tris, 150 mM NaCl, pH 7.4), and then flash frozen. After suspension in 500 μL TBS buffer containing HALT protease inhibitor (2 μl) and Superase RNase inhibitor (5 μl), cells were macerated with 0.1 mm glass beads (equal volume) by vortexing for 10 min with alternating 30 s intervals of vortexing and resting on ice. Cell debris was subsequently removed by centrifugation (18,000 x g for 30 min). After additional Superase inhibitor (5 μl) was added to the cleared lysate and the volume was brought up to 1 mL with TBS buffer, a portion of each sample was reserved for RNA and protein isolation (Input). The remaining sample was subsequently incubated with 75 μL of equilibrated anti-FLAG M2 resin for 2 h at 4°C. After washing the resin four times with TBS, bound proteins were eluted by incubation for 30 min with 150 μg/ml 3xFLAG peptide in TBS. The elution (IP) and input samples were then mixed with a neutral phenol solution (25:24:1 phenol:chloroform:isoamyl alcohol, pH 6.7) by vortexing for 10 s, and the aqueous phase and organic phases were separated by centrifugation. RNA was alcohol precipitated from the aqueous phase, and protein was precipitated from the phenol fraction by addition of two volumes of ice-cold acetone. RNA and protein were suspended in DEPC-treated water and Laemmli buffer, respectively. Northern blots were performed as described below in Materials and Methods. Western blotting analysis was performed by fractionating the samples reserved for protein analysis on a 4% stacking 10% resolving SDS-PAGE gel in 1 × Tris-glycine SDS buffer at 120 V. Fractionated protein was then transferred to a 0.45 μm PVDF membrane (Thermo Scientific) at 15 V for 30 min using Trans-Blot SD semidry transfer apparatus (Bio-rad) following manufacturer’s guidelines. PNPase was detected using 1:1000 dilution of a rat anti-FLAG antibody (1:1000) and anti-rat goat IgG secondary antibody (1:2500). Hfq was detected using 1:5000 dilution of preabsorbed anti-Hfq antiserum obtained from Susan Gottesman (NCI) and goat antirabbit IgG secondary antibody.

#### RNA stability assay

Overnight cultures were diluted 1:200 in fresh LB medium, grown to early exponential phase, and RyhB or CyaR were induced for 15 min by the addition of bipyridyl (250 μM) or cAMP (5 mM), respectively. 700 μL samples were taken from each culture (T = 0), rifampicin (250 μg/ml) was added to each culture, and additional samples were taken 1, 2, 5, and 10 min afterward. RNA was isolated by the hot phenol method as previously described (*39*).

#### Northern blot analysis

Unless indicated otherwise, 2 μg of each RNA sample was fractionated on 10% Tris-Borate-EDTA (TBE) urea gels by electrophoresis at 55V. RNA was then transferred to a Zeta-probe membrane (Bio-Rad) using the Trans-Blot SD semidry transfer apparatus (Bio-Rad) per manufacturer’s instructions. RNA was UV-crosslinked to the membrane and probed for each given RNA using a 5′ biotinylated DNA oligo of complementary sequence in ULTRAhyb (Ambion) hybridization buffer at 42°C. Blots were developed using the Ambion Brightstar BioDetect kit protocol. Chemiluminescent signals were detected using the ChemiDoc MP imager (Bio-Rad) and image analysis was performed using the Image Lab software v. 6.0.1. Signal intensity for each sRNA was normalized to a control RNA (SsrA). RNA turnover curves and half-lives were generated using GraphPad Prism 8.

#### Electron cryo-microscopy

##### Preparation cryo-EM samples supplemented with CHAPSO

PNPase, PNPase-sRNA and PNPase-sRNA-Hfq samples were prepared at 8-12 μM in a buffer not supporting catalysis, containing 20 mM Tris-HCl pH 8, 2.5 mM MgCl_2_, 150 mM KCl and 1 mM TCEP. 3 μl of each sample was applied to glow discharged (EasiGlow Pelco R2/2 Au Ultrafoil grids (Quantifoil)). Excess sample was blotted away with an FEI Vitrobot (IV) (100% humidity, 4°C, blotting force ranging from −2 to +2, 3 s blot time) immediately after addition of 8 mM CHAPSO (3-([3-cholamidopropyl]dimethylammonio)-2-hydroxy-1-propanesulfonate). CHAPSO was necessary to prevent the PNPase assemblies from adhering to the air-water interface during grid preparation. After blotting, the grids were vitrified in liquid ethane and screened on a FEI Talos Arctica. All datasets were collected on a FEI Titan Krios equipped with a Gatan K2 camera and the data collection parameters for all specimens are summarized in Table 1.

##### Image processing

For apo-PNPase and the PNPase-sRNA constructs, all processing steps were performed in Relion 3.0 (Scheres, 2012; Zivanov et al., 2018) and cryoSPARC (Punjani et al., 2017). In the Relion 3.0 analysis, a particle subset was manually selected to calculate reference-free 2D class averages, which were then used as templates for automated particle picking of the entire dataset. Several rounds of 2D and 3D classification were used to remove aberrant particles and false positive picks from the auto-picking. For 3D classification, an initial reference was generated in Relion via a stochastic gradient descent algorithm. Subsequent 3D auto-refinements resulted in high-resolution maps for which the PNPase core could be readily interpreted, after which per particle ctf refinement and per-particle motion correction/per frame radiation-damage weighting were carried out, followed by a final 3D refinement step. The final resolutions of the maps were estimated by 0.143 cut-off of the FSC and Local resolution variations were estimated in Relion using the two independent half-maps.

For apo-PNPase the particles were then subjected to C3 symmetry expansion. The expanded particle set was subjected to focused/masked classification without alignments to better resolve the KH-S1 portal. In parallel polished particles were transferred to cryoSPARC 2.15 and subjected to symmetry expansion and subsequent 3D variability analysis (3DVA) (6 modes, 6Å resolution limit). A schematic overview of the processing pipeline for apo-PNPase is displayed in Figure S1, and data processing is summarized in Table 1.

A schematic overview of the processing pipeline for PNPase-Hfq-3ʹETS^leuZ^ is displayed in Figure S3. Four different datasets, of which two with Volta Phase Plate (VPP), were collected and processed in Relion as described above. For each dataset the polished/ctf refined particle stacks were transferred to cryoSPARC for further rounds of heterogeneous refinement. Three discrete states were resolved and refined in CryoSPARC, the best of which refined to a global resolution of 3.7Å (GS-FSC). In parallel, the same strategy was used to resolve discrete sub-states of the KH-S1-Hfq region of the complex for the VPP dataset only. A set of particles for each of the two main conformational sub-states was used for 3DVA in cryoSPARC (6 modes, 6Å resolution limit). The PNPase-Hfq-CyaR dataset was processed in Relion 3.0 as described above.

##### Model docking and refinement

For initial docking into the apo-PNPase cryo-EM map, MODELER (Fiser and Šali, 2003) was used to generate a homology model based on the crystal structure of *Caulobacter crescentus* PNPase (pdb-ID 4aim) (Hardwick et al., 2012). In the latter the S1 and KH domains were resolved. Rigid body docking was performed in UCSF Chimera (Pettersen et al., 2004). An automated molecular dynamics-based approach, Namdinator (Kidmose et al., 2019), was used to further improve the global fit of the KH-S1 domains. This apo-PNPase model was then used as a starting model for fitting against the PNPase-sRNA and PNPase-Hfq-3ʹETS^leuZ^ /PNPase-Hfq-CyaR cryo-EM maps. For the PNPase sRNA maps, a co-crystal structure of *C. crescentus* PNPase bound to substrate RNA was used as a reference for the RNA backbone (pdb-ID 4am3) (Hardwick et al., 2012). For the ternary complexes, a crystal structure for *E. coli* Hfq was docked in the cryo-EM maps (pdb-ID 1HK9). The 3ʹETS^leuZ^ RNA backbone was traced in Coot (Emsley et al., 2010). Ramachandran outliers were fixed in Coot, as well as the overall geometry. Models were refined with Refmac5 as part of ccpem 1.4.1 (Murshudov et al., 2011; Burnley et al., 2017) and ISOLDE (Croll, 2018). The quality of the stereochemistry was evaluated via the comprehensive validation tool in Phenix 1.18 (Afonine et al., 2018), which uses molprobity (Chen et al., 2010). Images and movies were made in Chimera and PyMoL (DeLano, 2002; Pettersen et al., 2004). The statistics for model refinement are summarized in Table 1.

### Quantification and statistical analysis

Northern Blots (Figure 5) were quantified by detection of chemiluminescent signal (ChemiDoc MP imager, Bio-Rad) and the image analysis was performed using the Image Lab software v. 6.0.1. Signal intensity for each sRNA was normalized to a control RNA (SsrA). RNA turnover curves and half-lives were generated using GraphPad Prism 8, and represent the mean and standard error.

RNA degradation was quantified by recording of the fluorescence of RNA bands resulting from RNA degradation by PNPase/RNase E/RNase III on the urea PAA gel (GeneSys and GeneTools, Syngene) and comparing it to the internal standard. RNA turnover curves based on the mean and standard deviation of 3 independent assays were generated using GraphPad Prism 8.

## Supplemental information

Document S1. Figures S1–S6 and Tables S1–S3

Document S2. Article plus supplemental information
