# Supplementary material for: A cooperative PNPase-Hfq-RNA carrier complex facilitates bacterial riboregulation
Source: Mol Cell. 2021 Jul 15;81(14):2901–2913.e5. doi: 10.1016/j.molcel.2021.05.032 (PMC8294330; doi:10.1016/j.molcel.2021.05.032)
Supplement: Document S1. Figures S1–S6 and Tables S1–S3 [file mmc1.pdf]

Molecular Cell, Volume *81*

Supplemental information

A cooperative PNPase-Hfq-RNA carrier complex facilitates bacterial riboregulation

Tom Dendooven, Dhriti Sinha, Alzbeta Roeselová, Todd A. Cameron, Nicholas R. De Lay, Ben F. Luisi, and Katarzyna J. Bandyra

Figure S1. Sample optimisation, global resolution estimates according to GS-FSC for the three species presented in this manuscript and schematic overview of the processing pipeline for apo-PNPase. Related to Figures 1 and 2.

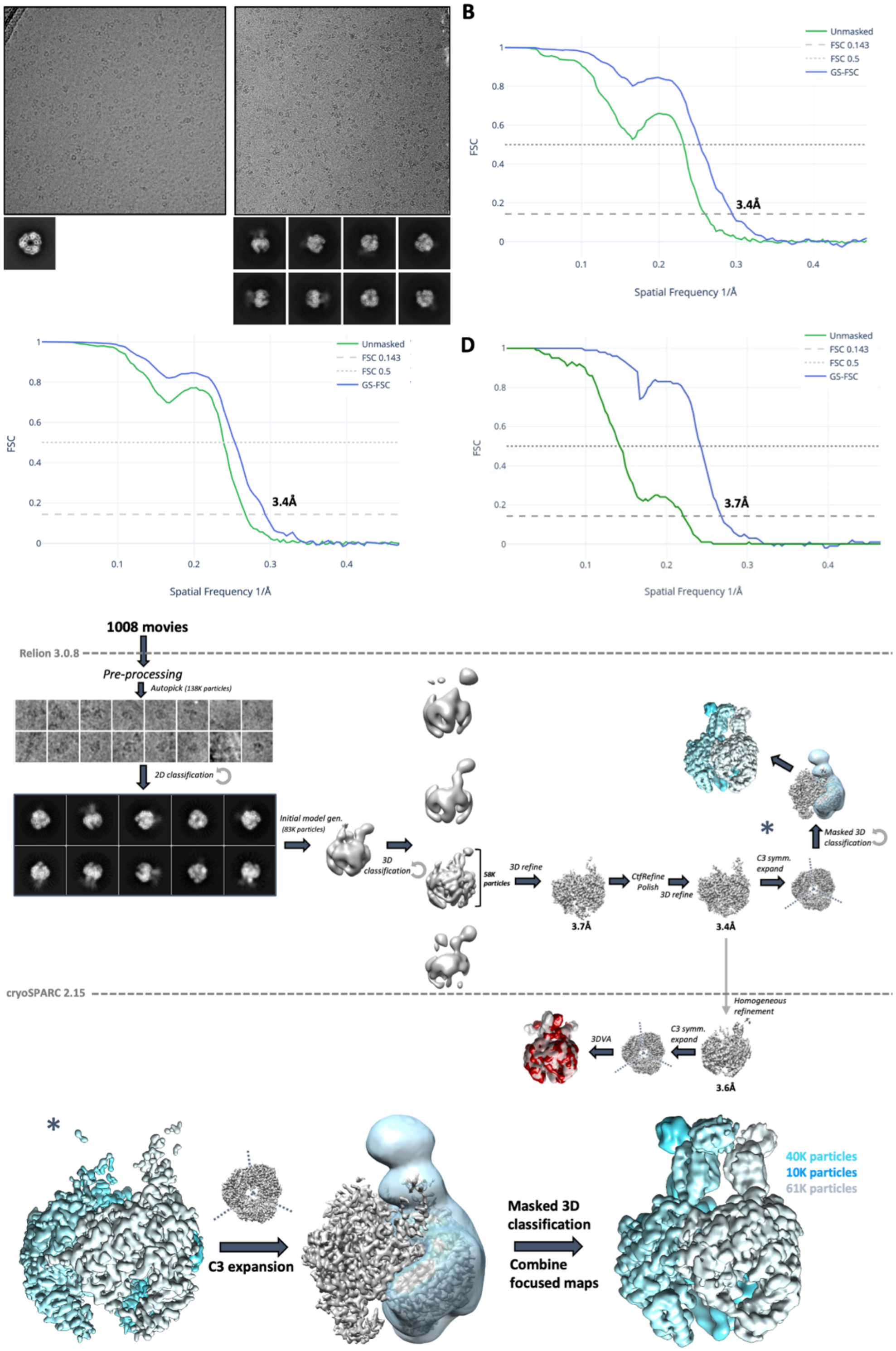

(**A**). Representative cryo-EM images (2.5mm defocus) and 2D class averages before (left) and after (right) adding 8 mM CHAPSO to the sample. In the absence of CHAPSO the PNPase particles adhere to the air-water interface and

adopt preferred orientations in the vitreous ice. (**B-D)**. FSC curves for apo-PNPase, PNPase-3′ETS^{leuZ} and PNPase-Hfq-3′ETS^{leuZ}, respectively, as calculated from independent half-maps. **(E).** Pre-processing, which entails motion correction, ctf estimation, manual picking and 2D reference generation was carried out in Relion 3.0.8. Consensus maps were refined to 3.4Å (GS-FSC) prior to C3 symmetry expansion and focused 3D classifications (see * in figure, further explained in **F**). In parallel, a clean particle set was transferred to CryoSPARC for symmetry expansion and subsequent 3D variability analysis (Punjani and Fleet, 2020). (F**)**. The symmetric PNPase core drives the particle alignments so that the flexible KH and S1 domains are averaged out in consensus reconstructions. Using symmetry expansion tools and masked 3D classification in Relion 3.0, KH-S1 portal was resolved and three conformational states were found (Zivanov *et al.*, 2018). In these sub-states, the resolution of the KH domains improved significantly, and the density for the S1 domains was also enhanced. A combined map was generated from three focused 3D classes and colour coded (right). Particle numbers contributing to each conformational state are depicted on the right. These improvements in the cryo-EM map allowed for local rigid-body docking and molecular dynamics fitting of the KH and S1 domains with Namdinator and coot (Emsley *et al.*, 2010; Kidmose e*t al.,* 2019).

Figure S2. Cryo-EM analysis of the PNPase-Hfq-3′ETS^{leuZ} and PNPase-Hfq-CyaR complexes. Related to Figures 2 and 4.

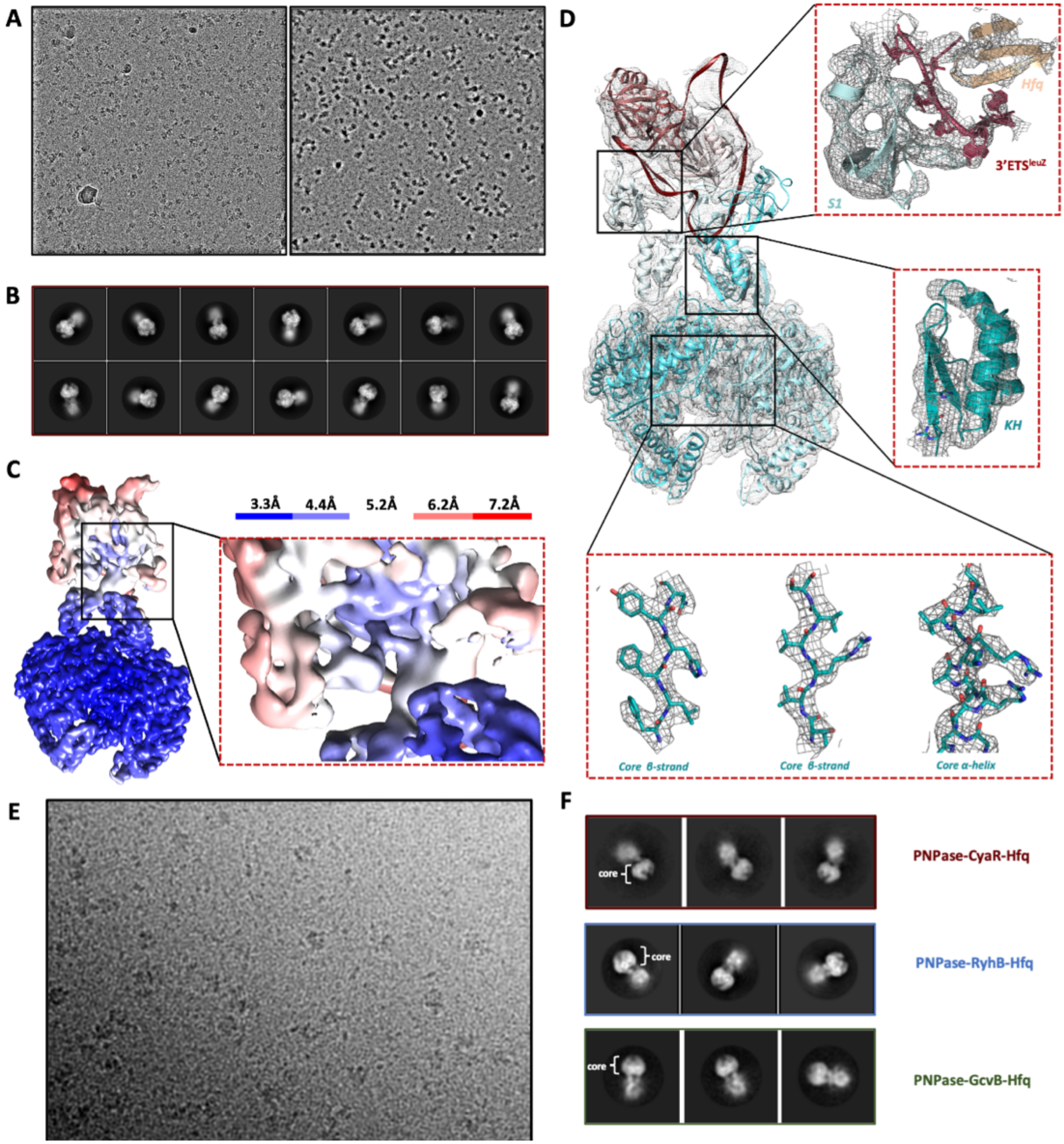

(**A**). Representative micrographs without Volta Phase Plate, VPP, (left, 3 μM defocus) and with VPP (right 0.5 μM defocus). (**B**). 2D class averages show a well resolved core and a diffuse KH/S1/Hfq portal. (**C**). Local resolution estimation and filtering as performed in cryoSPARC. The PNPase core is rigid and can be reconstructed at 3.2Å. The KH, S1, 3′ETS^{leuZ} and Hfq densities suffer from a loss in resolution due to severe flexibility, with local resolutions ranging from 4.4 to 7.2Å. Local resolutions were calculated from reconstructed half maps, at FSC 0.5. (**D**). Selected regions of the model and corresponding cryo-EM density at the PNPase core and KH/S1/Hfq portal. (**E**). Representative cryo-EM image of PNPase-Hfq -CyaR at 2.5 μm defocus.

(**F**). Selected 2D class averages for PNPase-CyaR-Hfq, PNPase-RyhB-Hfq and PNPase-GcvB-Hfq generated in cryoSPARC reveal diffuse yet bulky densities at the KH-S1 portal. The overall quaternary architecture of all three RNA carrier complexes is similar to the PNPase-3'ETS^{leuZ}-Hfq assembly. The PNPase core is annotated in the 2D classes averages.

Figure S3. Schematic overview of the processing pipeline for PNPase-Hfq-3′ETS^{leuZ}. Related to Figure 2.

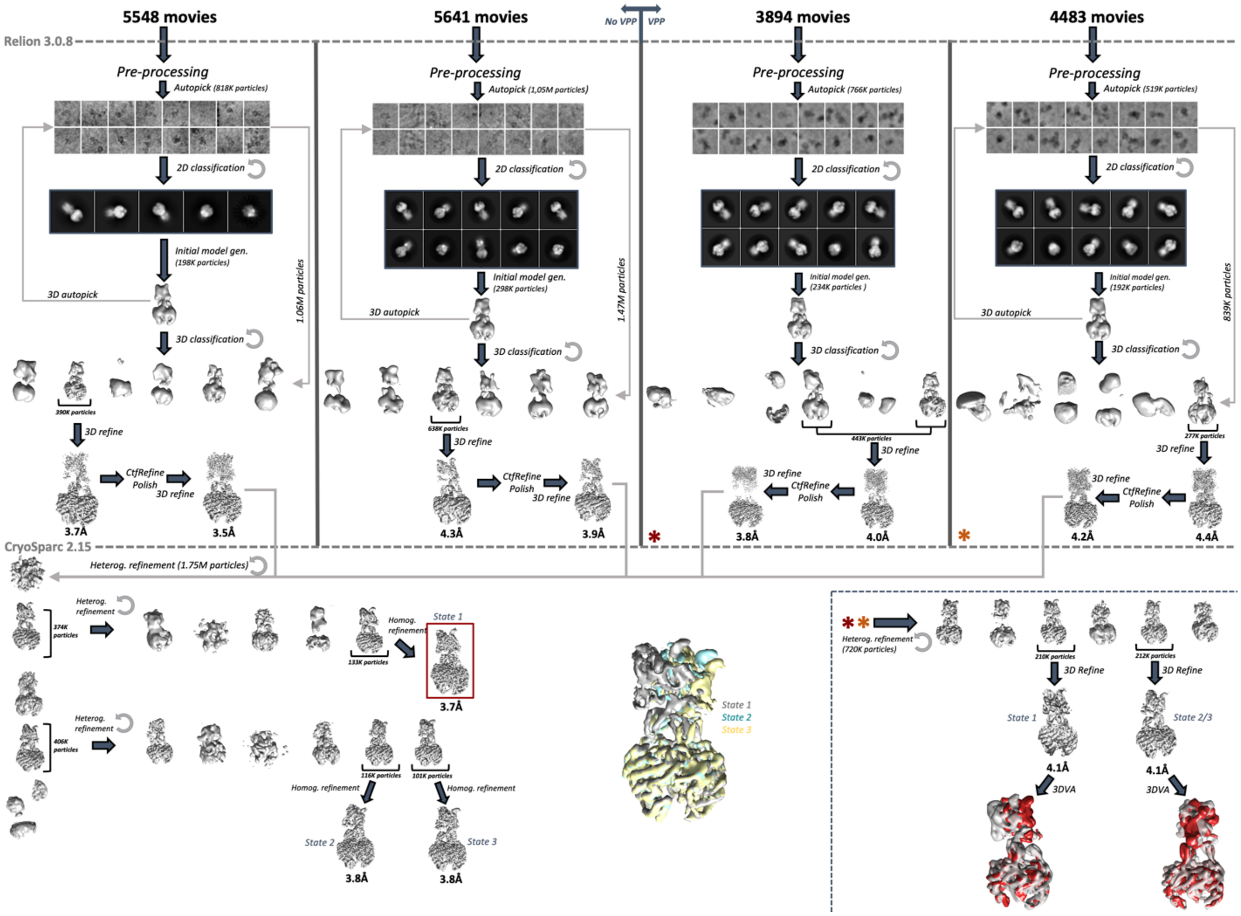

For all four datasets pre-processing, which entails motion correction, ctf estimation, manual picking and 2D reference generation was carried out in Relion 3.0.8. 2D/3D classifications were carried out in Relion, as well as consensus refinements and per particle ctf refinement/ particles polishing. Clean particle sets for each dataset we transferred to cryoSPARC and combined. Subsequent rounds of heterogenous refinements were used to resolve three different conformational states (state 1, state 2, and state 3), each of which refined to sub-4Å resolutions. In parallel, conformational heterogeneity was also resolved through heterogeneous refinement for the particles sets from the VPP-datasets (see *, bottom right inset). To further analyse residual conformational heterogeneity for each state, 3D variability analysis was carried out.

Figure S4. Conformational heterogeneity of the KH-S1 portal. Related to Figures 1 and 2.

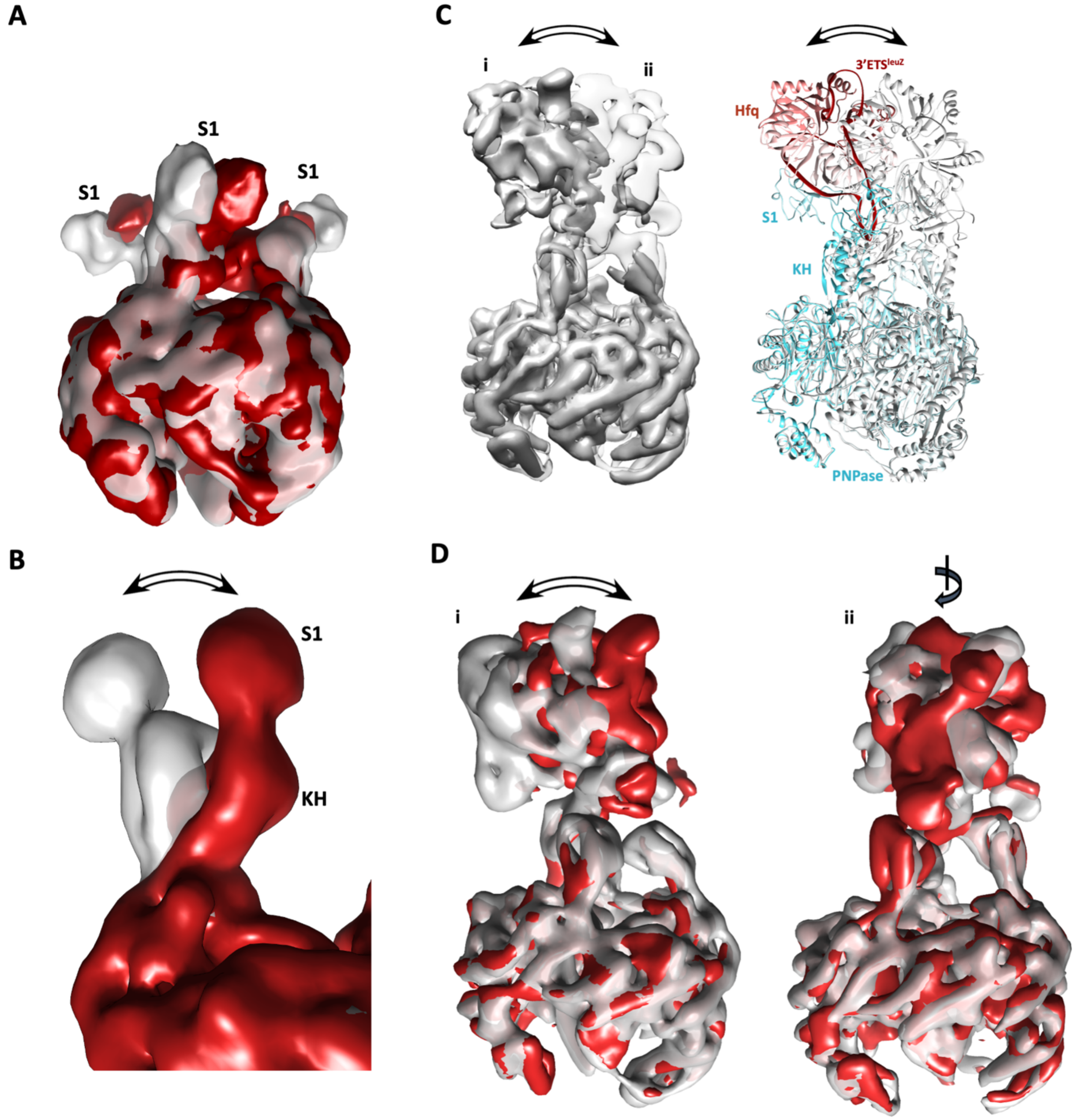

(A). cryoSPARC 3D variability analysis of apo-PNPase after C3 symmetry expansion (Punjani *et al.*, 2017; Punjani and Fleet, 2020). Grey and red maps represent extreme states of the molecular motion of the KH and S1 domains as encoded in the dominant 3DVA mode. (**B**). Zoom-in on one KH-S1 region, showing conformational heterogeneity of these domains as encoded in the dominant 3DVA mode. (**C**)**.** The PNPase-Hfq-3′ETS^{leuZ} assembly can adopt multiple conformations along a continuous clapper-like trajectory, as resolved here by heterogeneous refinement in cryoSPARC (i and ii are two extreme conformations). A similar set of conformations was resolved by 3D classification in Relion (data not shown). (**D**)**.** Particles corresponding to state i and ii from panel **C** were refined separately and subjected to 3DVA in cryoSPARC to reveal residual conformational heterogeneity. This approach resulted in a more

modest ‘rocking’ mode for i and a ‘rotary’ mode for ii. All maps presented here are lowpass filtered to 8Å for visibility.

Figure S5. PNPase WT and mutants (S1x2, KHx2 and S1x4) display different levels of activity towards sRNAs. Related to Figures 3, 5 and 6.

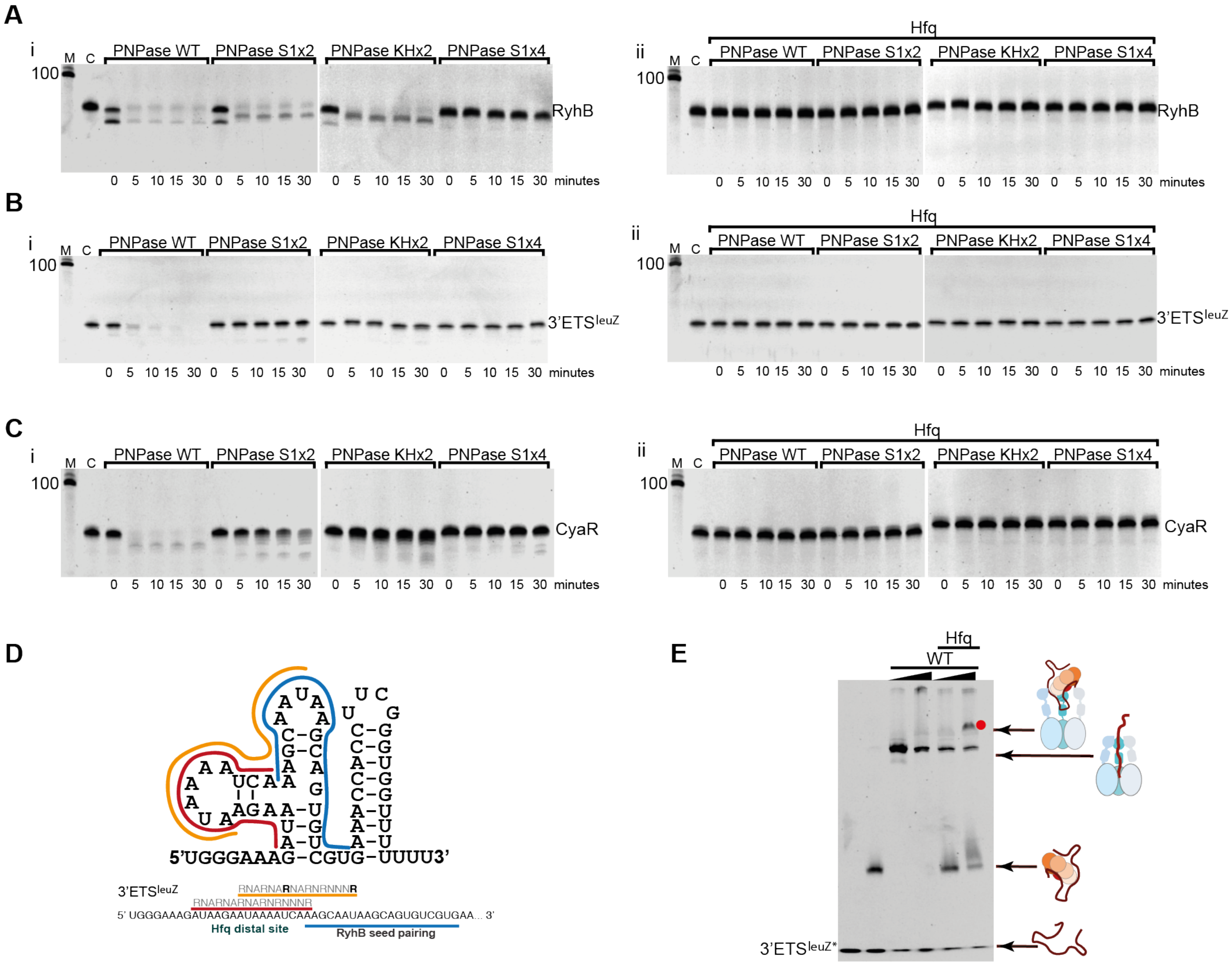

Degradation of 200 nM RNAs: RyhB (**A**), 3′ETS^{leuZ} (**B**) and CyaR (**C**) by 200 nM PNPase trimer in the absence (i) or presence (ii) of 200 nM Hfq hexamer. M is the molecular weight marker, C is control RNA without PNPase treatment. Different PNPase degradation products can result from differences in RNA binding and/or different speed of phosphorolysis of the wild type and mutant enzymes. (**D**) Mapping of the 3′ETS^{leuZ} ARN-motifs that can bind to the Hfq distal side in the RNA carrier assemblies. Two putative RNARNARNARNRNNNR motifs were identified in the 5' half of 3′ETS^{leuZ} (red and orange). In the orange mapping two R sites on the Hfq distal side would be occupied by pyrimidine bases. The RyhB seed region is annotated in blue. (**E**) EMSA of wild type PNPase with 400 nM 3′ETS^{leuZ*} (processed version of 3′ETS^{leuZ}) in the absence and presence of 400 nM Hfq hexamer. Two different PNPase concentrations were used (1:1 and 1:3 RNA:PNPase trimer molar ratio), represented by a crescendo bar.

Figure S6. PNPase pull down after dipyridyl treatment. Related to Figure 1.

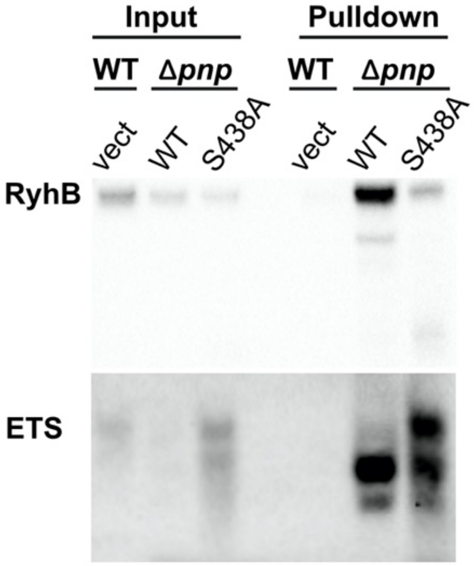

PNPase pull down after dipyridyl treatment, showing that both RyhB (top panel) and 3′ETS^{leuZ} (lower panel) are bound to PNPase. WT indicates wild type PNPase and S438A is an active site mutant. PNPase activity may be required to process 3′ETS^{leuZ}, and the decreased yield of RyhB for the inactive mutant is proposed to arise from its binding site being occluded by other RNA species that are trapped on the enzyme (see also Cameron *et al.* 2019, Figure 3 and Figure S3).

Table S1. Primers and oligonucleotides used in this study. Related to STAR Methods

Name	Sequence (5' to 3')
**Primers and oligonucleotides for IVT**	
chiP_F	GTTTTTTAATACGACTCACTATAGGTTTTTATTTTTTCGCTGTTCACCTTTGGTGCAGCAATTTATACGTCAAAGAGGATTAAC
chiP_R	GTTAATCCTCTTTGACGTATAAATTGCTGCACCAAAGGTGAACAGCGAAAAAATAAAAACCTATAGTGAGTCGTATTAAAAAAC
CyaR_F	GTTTTTTTTTTAATACGACTCACTATAGCTGAAAAACATAACCCATAAAATGCTAGCTGTACCAGGAACCACCTCCTTAGCCTGTGTAATCTCCCTTACACGGGCTTATTTTTT
CyaR_R	AAAAAATAAGCCCGTGTAAGGGAGATTACACAGGCTAAGGAGGTGGTTCCTGGTACAGCTAGCATTTTATGGGTTATGTTTTTCAGCTATAGTGAGTCGTATTAAAAAAAAAAC
ETS_F	GTTTTTTTAATACGACTCACTATAGGGAAAGATAAGAATAAAATCAAAGCAATAAGCAGTGTC GTGAAACCACCTTCGGGTGGTTTTTTT
ETS_R	AAAAAAACCACCCGAAGGTGGTTTCACGACACTGCTTATTGCTTTGATTTTATTCTTATCTTTCCCTATAGTGAGTCGTATTAAAAAAAC
GcvB_F	GTTTTTTTTTTAATACGACTCACTATA G ACTTCCTGAGCCGGAACGAAAAG
GcvB_R	AAAAAAAAGCACCGCAATTAGGCGGTG
ompX_F	GTTTTTTAATACGACTCACTATAGGACTTATTTGAATCACATTTGAGGTGGTTatgAAAAAAATTGCATGTCTTTCAGCACTG
ompX_R	CAGTGCTGAAAGACATGCAATTTTTTTcatAACCACCTCAAATGTGATTCAAATAAGTCCTATAGTGAGTCGTATTAAAAAAC
RyhB_F	tataGAATTCTAATACGACTCACTATAGCGATCAGGAAGACCCTC
RyhB_R	aaaaaaaaagccagcacccggctggctaag
**Primers for PCR**	
pnp For	GGGACGTCGGTTAGGGTTGTCATTAGTCG
pnpmidFor3	CGTACTCACGGTTCTGCG
pnp Rev	GGGACGTCGAATGAACGTCCTGTTCCC
pnpfar For	GCTTTACCCACATAGAGCTGG
pnpfar Rev	CCACGGTTGAATGAACGTCC
pnpD566-71KO For	ACCGCGTATCCATACCATCAAGATCAACCCGGACAAGATCTTATATTCCCCAGAACATCAGG
pnpD566-71KO Rev	TGCCAGTTTCTTCGGTCAGAGCACGGATTACAGAACCGCCATAGGAACTTCAAGATCC
pnpD657-8KO For	CGGTAAAGAAGGTCTGGTCCACATCTCTCAAATCGCTGACTTATATTCCCCAGAACATCAGG
pnpD657-8KO Rev	CTTCCTGACCCATCTGCAGGTAATCGGTCACTTTCTCAACATAGGAACTTCAAGATCC
pnpD681-6KO For	GATGGGTCAGGAAGTACCGGTGAAAGTTCTGGAAGTTGATTTATATTCCCCAGAACATCAGG
pnpD681-6KO Rev	CAGCAGGTTGAGACTGCTCAGTCGCTTCTTTAATGCTCAGATAGGAACTTCAAGATCC
pnpK566K571	CCATACCATCAAGATCAACCCGGACAAGATCGCAGATGTTATCGGTGCAGGCGGTTCTGTAATCCGTGCTCTGACCGAA
pnpK657R658	AAGAAGGTCTGGTCCACATCTCTCAAATCGCTGACGCCGCTGTTGAGAAAGTGACCGATTACCTGCAGATGGGTCA
pnpRQRR681-6	GAAGTACCGGTGAAAGTTCTGGAAGTTGATGCTGCAGGCGCAATCGCTCTGAGCATTAAAGAAGCGACTGAGCAGTCT
PNPaseNcoFor	TATACCATGGTTGCTTAATCCGATCGTTCGTAAATTCC
PNPaseNotRev	TATATAGCGGCCGCTTACTCGCCCTGTTCAGCAGCCG
PNPaseS1x2For	CTCTCAAATCGCTGACGCGGCGGTTGAGAAAGTGACCG
PNPaseS1x2Rev	CGGTCACTTTCTCAACCGCCGCGTCAGCGATTTGAGAG
PNPaseKHx2For	CAACCCGGACAAGATCGCGGATGTTATCGGTGCGGGCGGTTCTGTAATCC
PNPaseKHx2Rev	GGATTACAGAACCGCCCGCACCGATAACATCCGCGATCTTGTCCGGGTTG
PNPaseS1x4For	GTTCTGGAAGTTGATGCGGCGGGCGCGATCGCGCTGAGCATTAAAGAAG
PNPaseS1x4Rev	CTTCTTTAATGCTCAGCGCGATCGCGCCCGCCGCATCAACTTCCAGAAC
**Probes**	
CyaR	TGGTTCCTGGTACAGCTAGCATTTTATGGGTTATG
ETS	CCACCCGAAGGTGGTTTCACGACACTGCTTATTGCTTTG
RyhB	AAGTAATACTGGAAGCAATGTGAGCAATGTCGTGCTTTCAGGTTCTC
SsrA	CGCCACTAACAAACTAGCCTGATTAAGTTTTAACGCTTCA

Table S2. sRNA Half-lives Related to Figure 5.

Strain	Average half-life (min) ± SE	sRNA
NRD1243 *pnp'-3xFLAG*	17.5 ± 4.2	CyaR
NRD1369 *Δpnp*	6.8 ± 1.9	
NRD1614 *pnp(2x2)'-3xFLAG*	8.1 ± 1.5	
NRD1615 *pnp(2x1)'-3xFLAG*	12.7 ± 1.9	
NRD1622 *pnp(4x)'-3xFLAG*	9.8 ± 1.3	
NRD1243 *pnp'-3xFLAG*	14.0 ± 2.7	RyhB
NRD1369 *Δpnp*	2.5 ± 0.72	
NRD1614 *pnp(2x2)'-3xFLAG*	4.1 ± 0.90	
NRD1615 *pnp(2x1)'-3xFLAG*	4.1 ± 1.1	
NRD1622^{+} *pnp(4x)'-3xFLAG*	6.5 ± 1.1	
NRD1243 *pnp'-3xFLAG*	1.6 ± 0.18	$3'ETS^{leuZ}$
NRD1369 *Δpnp*	1.1 ± 0.11	
NRD1614 *pnp(2x2)'-3xFLAG*	1.5 ± 0.13	
NRD1615 *pnp(2x1)'-3xFLAG*	1.2 ± 0.08	
NRD1622 $^{+}$ *pnp(4x)'-3xFLAG*	1.1 ± 0.11	

Table S3. Strains and plasmids used in this study. Related to STAR Methods

Strain or plasmid	Relevant features	Origin
**Strains**		
BL21(DE3)	(λDE3)	
KR10000	MG1655 *rph*$^+$	D. Court, NCI
NRD999	MG1655 *rph*$^+$ Δ*pnp::cat*	Bandyra 2016
NRD1243	MG1655 *rph*$^+$ *pnp'-3xFLAG*	Cameron 2016
NRD1369	MG1655 *rph*$^+$ Δ*pnp*	Cameron 2016
NRD1611	MG1655 *rph*$^+$ *pnp*Δ*566-71::ccdB kan::3xFLAG*	This study
NRD1612	MG1655 *rph*$^+$ *pnp*Δ*657-8::ccdB kan::3xFLAG*	This study
NRD1613	MG1655 *rph*$^+$ *pnp*Δ*681-6::ccdB kan::3xFLAG*	This study
NRD1614	MG1655 *rph*$^+$ *pnp(2x2)'-3xFLAG*	This study
NRD1615	MG1655 *rph*$^+$ *pnp(2x1)'-3xFLAG*	This study
NRD1617	BL21(DE3) Δ*pnp::cat*	This study
NRD1622	MG1655 *rph*$^+$ *pnp(4x)'-3xFLAG*	This study
CR201	*cya::kan ccdB*	C. Ranquet
NRD1137	MG1655 *rph*$^+$ Δ*pnp::cat rne-131 zce-726:*Tn*10*	Cameron 2019
**Plasmids**		
pKD46	Amp^R, RepA101(Ts), λ γ, β, and *exo* expressed from an *araBAD* promoter	Datsenko 2000
pTC352	Amp^R; P_{lac} *araB-5'UTR pnp'-3xFLAG lacI*^{q}	Cameron 2019
pTC354	Amp^R; P_{lac} *araB-5'UTR pnp(S438A)'-3xFLAG lacI*^{q}	Cameron 2019
pTC396	Amp^R; P_{lac} *lacI*^{q}	Cameron 2019
